# A Coupled Model of Acid Transport, Gel Cleanup, and Fracture Propagation in Prepad Acid Fracturing

**DOI:** 10.3390/gels12070622

**Published:** 2026-07-10

**Authors:** Weiyou Zhang, Yongpeng Sun, Xianghua Meng, Rutong Dou

**Affiliations:** 1 College of Energy and Mechanical Engineering, Dezhou University, Dezhou 253023, China; mengxianghuadz@163.com (X.M.); dourutong@163.com (R.D.); 2 Dezhou Huahai Petroleum Machinery Co., Ltd., Dezhou 253000, China; 3 Shandong Ruiyuan Petroleum Technology Co., Ltd., Dezhou 253000, China; 4 School of Petroleum Engineering, China University of Petroleum (East China), Qingdao 266580, China; sunyongpeng@upc.edu.cn

**Keywords:** prepad acid fracturing, gel filter cake cleanup, fluoroboric acid, coupled simulation, stimulation performance

## Abstract

In conventional hydraulic fracturing of low-permeability sandstone reservoirs, polymer-gel leak-off creates low-permeability filter cakes that impair productivity. This study proposes a prepad acid fracturing technique using a fluoroboric acid (HBF_4_) pre-flush to dissolve gel residues and mineral fines. A fully coupled mathematical model integrates HBF_4_ hydrolysis kinetics, multi-mineral surface reactions, porosity-permeability evolution via the Panda–Lake model, and dynamic leak-off coefficient feedback. Simulations show HBF_4_ decreases monotonically along the fracture while HF peaks at 40–60 m from wellbore. Acid concentration in the leak-off zone decays exponentially, defining a gel-dissolution zone within 0.5 m of the fracture wall. Acid dissolution increases near-wall porosity to 12–15% and permeability to 2.5–3.5 mD (3- to 4-fold). The leak-off coefficient varies dynamically: high in the acid-dominated zone (1.5–2.2 × 10^−3^ m/√min) favoring gel dissolution, and low in the gel-dominated zone (≈0.8 × 10^−3^ m/√min) promoting fracture extension. Compared with conventional polymer gel fracturing, the proposed method achieves a 15.9% higher stimulation ratio and 22.5% higher productivity after 100 days, despite slightly shorter fractures. The core advantage is restoring leak-off zone permeability from 0.45 mD to 0.85 mD and increasing gel filter cake permeability from 8 × 10^−4^ mD to 0.1 mD, with an average relative error of 8.2% against experimental data. These findings provide theoretical guidance for optimizing prepad acid fracturing in gel-damaged low-permeability sandstones.

## 1. Introduction

Polymer-based gel fluids are one of the most widely used fracturing fluids in low-permeability reservoirs. However, reservoir damage induced by the leak-off of these viscous gel systems has long constrained the achievable stimulation efficiency. During fracturing operations, the leak-off of polymer gel fluids into the rock matrix outside the fracture face can cause multiple damage mechanisms: pore throat plugging by polymer residues and gel fragments, clay mineral swelling due to incompatible fluids, and the formation of a low-permeability gel filter cake on the fracture wall. These mechanisms collectively reduce fracture conductivity and impair long-term productivity [1,2,3]. In sandstone reservoirs in particular, the interaction between residual polymers and clay minerals may lead to irreversible permeability impairment [4]. Consequently, mitigating leak-off zone damage—especially removal of the gel filter cake—while maintaining effective fracture propagation remains a critical scientific challenge in reservoir stimulation.

Prepad acid fracturing involves injecting an acid pre-flush ahead of the subsequent polymer gel fracturing fluid stages [5,6]. The acid that leaks off into the formation during this pre-flush stage reacts with rock minerals and any residual gel components present in the near-wellbore region, dissolving bridging particles and cleaning pore throats, thereby restoring reservoir permeability. In addition, as the polymer gel fracturing fluid is injected later and forms a filter cake on the fracture wall, the acid that remains or is transported ahead of the fracturing fluid can interact with this gel filter cake, partially breaking the polymer network and non-uniformly etching the fracture wall to form self-supporting flow channels after fracture closure, while also reducing near-wellbore flow resistance [7,8]. Recent field applications have demonstrated that prepad acid fracturing achieves significant stimulation improvements in both low-permeability sandstone and carbonate reservoirs, with stimulation ratios 15–30% higher than those of conventional polymer gel fracturing [9,10].

Regarding acid system selection for gel damage removal, fluoroboric acid (HBF_4_) has attracted particular attention due to its unique hydrolysis characteristics. HBF_4_ slowly hydrolyzes in aqueous solution to generate hydrofluoric acid (HF), theoretically enabling both deep penetration into the leak-off zone and a retarded reaction rate to prevent premature acid spending [11]. Experimental studies on the reaction of kaolinite with HBF_4_ have shown that HBF_4_ exhibits a “strong acid but delayed” behavior, with significant dissolution of alum inosilicate minerals occurring only after 0.5–1 h of reaction, whereas conventional mud acid (HCl/HF) reacts much more rapidly [12]. However, classic work by Kunze and Shaughnessy [13] indicates that under typical reservoir temperature conditions, the hydrolysis rate of HBF_4_ accelerates significantly, and its activity decay rate becomes comparable to that of conventional HF, potentially weakening its deep-penetration advantage. Moreover, the HBF_4_ system still faces the risk of secondary precipitation, especially the formation of fluorosilicates and fluoroaluminates, which may cause additional formation damage [14]. While these precipitation reactions are recognized as important considerations in acid fracturing design, their inclusion in the current model would require additional kinetic parameters and species transport equations that are not yet well-constrained by experimental data for the specific conditions of this study. The present model therefore focuses on the primary acid–rock dissolution reactions that govern gel filter cake cleanup and permeability enhancement, while acknowledging secondary precipitation as a potential limitation that should be addressed in future model extensions.

The mathematical description of acid fracturing has evolved from early analytical models to multi-physics coupled numerical models. Early acid fracturing models primarily focused on the relationship between fracture geometry and effective acid penetration distance, using average reaction rate assumptions that cannot capture the coupling between acid leak-off and porosity–permeability evolution [15]. In recent years, with the development of computational fluid dynamics and reactive transport theory, various numerical simulation methods for acid fracturing have been proposed. Panga et al. [16] developed a two-scale continuum model coupling Darcy-scale and pore-scale processes, enabling simulation of wormhole propagation during acidizing. Yang et al. [17] investigated acid leak-off behavior using an improved dual-scale continuous model, revealing the effects of injection rate, acid viscosity, and concentration on the leak-off coefficient. Regarding the impact of acid pre-treatment on hydraulic fracturing, Yang et al. [18] established a hydro mechanical–chemical multi-field coupled model and found that acid pre-treatment significantly reduces breakdown pressure, with acid concentration being the key factor controlling the peak pressure reduction. Li et al. [19] studied the cleanup mechanism of micro emulsion acid using microfluidic experiments, revealing the microscopic mechanism by which surfactants reduce capillary pressure and promote acid flowback.

From the perspective of gel-based fracturing fluids, the interaction between acid pre-flushes and polymer gel filter cakes remains underexplored in existing models. Most studies treat the gel filter cake as an impermeable barrier with constant properties, ignoring its potential modification by acid contact [20,21,22]. Furthermore, numerical simulation studies specifically targeting prepad acid (HBF_4_) in the context of polymer gel damage removal are relatively scarce, and a complete model accounting for HBF_4_ hydrolysis to HF, multi-mineral reactions, gel filter cake alteration, and porosity–permeability coupling is lacking [23,24]. The dynamic variation of the leak-off coefficient—especially the transition from acid-dominated to gel-dominated leak-off—has not been systematically characterized, making it difficult to guide optimal design of acid volume and injection rate [25]. This gap underscores the need for a coupled modeling approach that explicitly links acid transport, gel alteration, and leak-off dynamics.

The dynamic evolution of filter cake pore structure during pressure-driven dewatering has been extensively investigated in recent years. Ma et al. [26] conducted a quantitative analysis of high-efficiency dewatering under ultrahigh pressures using low-field nuclear magnetic resonance (LF-NMR) relaxation time T_2_ and computed tomography (CT) scanning to characterize the moisture storage state and pore structure evolution of filter cakes. Their experimental results demonstrated that, as pressure increases from 1 MPa to 5 MPa, the filter cake undergoes progressive pore collapse: at pressures below 1 MPa, the cake is more compressible with longer and wider permeation paths; at 3 MPa, pores begin collapsing and shrinking into mesopores and micropores while maintaining good connectivity; at 4 MPa, channel seepage transitions into microporous seepage with decreased dehydration efficiency; and at 5 MPa, most micropores are compressed and closed, resulting in minimal cake moisture [26]. These findings provide direct experimental evidence that filter cake pore structure—and thus permeability—evolves significantly with pressure and dewatering conditions, underscoring the importance of capturing such dynamic behavior in models of gel filter cake cleanup.

In addition to permeability enhancement, acid-induced mineral dissolution can significantly alter the mechanical integrity of the rock matrix, which has important implications for long-term fracture conductivity stability. Li et al. [27] systematically investigated the mechanical properties of sandstones damaged by CO_2_ reactions and their flow characteristics under complex mixed-wettability conditions. Their experimental results demonstrated that the acid production reaction between CO_2_ and water in rocks changes the pore structure, thereby altering the rock mechanical properties [27]. Under complex mixed-wettability conditions, the displacement efficiencies of gaseous CO_2_ and supercritical CO_2_ decreased by 5.7% and 15.3%, respectively, compared with those observed under single-phase wettability, indicating that wettability heterogeneity significantly influences fluid transport in chemically altered porous media [27]. These findings highlight that fluid–rock reactions not only enhance permeability through mineral dissolution but may also weaken the mechanical strength of the rock skeleton, potentially affecting the stability of fracture conductivity under in-situ stress conditions. While the present study focuses on the beneficial effects of acid dissolution on permeability restoration, the potential for mechanical weakening of the near-fracture-wall region due to mineral removal should be considered when evaluating long-term fracture conductivity stability, particularly in reservoirs with weak cementation or high clay content.

Recent advances in acid fracturing technology have also explored novel acid systems and their interactions with reservoir rocks. Chen et al. [28] investigated acid dissolution patterns along rough-walled fractures with effects of mineral compositions and fracture geometries, demonstrating that increasing mismatch length concentrates acid flow, decreases effective dissolution volume, and enhances channel conductivity [28]. Their numerical simulations revealed that fracture heterogeneity significantly affects dissolution morphology, providing important insights for optimizing acid fracturing designs in heterogeneous formations. Additionally, Yang et al. developed and evaluated acid-resistant preformed particle gels for anti-CO_2_ channeling in high-temperature reservoirs, achieving a plugging rate of 93.4% in low-permeability micro-fractured cores [29]. The development of low-viscosity polymeric retarding agents with dual adsorption layers for acid fracturing has also been reported, addressing the limitations of acid penetration distance and acid-etched fracture conductivity [30]. These studies collectively demonstrate the ongoing evolution of acid fracturing technology and the importance of understanding acid–rock–gel interactions at multiple scales.

In recent years, peridynamics (PD) has emerged as a powerful numerical tool for simulating hydraulic fracture propagation in complex geological settings, overcoming the limitations of conventional continuum-based methods in handling discontinuities and fracture branching. Qin et al. [31] developed a three-dimensional hydraulic fracturing model based on peridynamics and validated it against analytical solutions, demonstrating that 3D models capture fracture height growth and HF–NF interactions that are unattainable in 2D modeling. More recently, peridynamics-based simulations have been extended to investigate hydraulic fracture interaction with natural fractures in three-dimensional fractured rock masses, successfully reproducing complex interaction patterns such as fracture ‘diversion’ and the propagation of intricate fracture networks in arbitrarily fractured media [32]. These studies have revealed that in-situ stress ratio, fluid viscosity, injection rate, and fracture approach angle collectively govern the transition between HF penetration into and diversion around natural fractures [32]. Additionally, peridynamics has been applied to simulate HF height growth in layered rocks [33], fracture propagation in heterogeneous rock masses [34], and hydro-mechanical coupling in fractured rock under dynamic loading [35].

Despite the demonstrated capabilities of peridynamics for capturing complex 3D fracture geometries, the computational cost of fully coupled 3D PD simulations remains prohibitively high for routine engineering design optimization, particularly when coupled with reactive transport and porosity–permeability evolution as in the present study. The primary objective of this work is to investigate the coupled acid transport, gel filter cake cleanup, and fracture propagation mechanisms under controlled reservoir conditions, where the reservoir is characterized as a relatively homogeneous low-permeability sandstone without pervasive natural fracture networks. Under such conditions, the classical PKN (Perkins–Kerr–Norden) or KGD (Khristianovich–Geertsma–de Klerk) fracture propagation framework provides a computationally efficient and physically adequate approximation for simulating the dynamic coupling between acid leak-off, gel cleanup, and fracture extension [36,37].

In addition to the aforementioned advances, recent studies have further expanded the understanding of acid fracturing performance through novel acid systems and enhanced characterization techniques. Al Drwish et al. [38] investigated the enhancement of fracture conductivity using CO_2_-foamed acid for elevated temperature wells, demonstrating that foamed acid systems can significantly improve acid penetration distance and etched fracture conductivity compared with conventional acid systems under high-temperature conditions. Zhang et al. [39] developed an autogenous solid acid for acid fracturing in deep carbonate reservoirs, achieving controlled acid release and sustained reactivity that addresses the limitations of rapid acid spending in high-temperature formations. Furthermore, Bai et al. [40] examined the effects and mechanism of gaseous acid on surface microtopography of tight sandstone reservoirs, revealing that gaseous acid treatment alters surface roughness and wettability, which in turn influences fluid flow characteristics and overall stimulation efficiency. These studies collectively underscore the importance of continued innovation in acid systems and the need for integrated characterization of acid–rock interactions across multiple scales. Together with the classic PKN [36] and KGD [37] fracture propagation frameworks, these recent advances provide a comprehensive foundation for the development of the coupled model presented in this work.

To address these gaps, this study develops a coupled mathematical model for acid concentration distribution in the fracture and the leak-off zone, based on acid–rock reaction kinetics and mass conservation principles. While previous studies have addressed individual components of this system—acid transport in fractures [16,17,18], gel filter cake damage [20,21,22], and porosity–permeability evolution [23,24]—none have simultaneously coupled these processes with HBF_4_ hydrolysis kinetics and real-time leak-off coefficient feedback in a unified framework. The key novelty of our model lies in this integration, which explicitly links: (i) the reaction kinetics of HBF_4_ hydrolysis to HF; (ii) surface reaction mechanisms for three mineral categories; (iii) dynamic evolution of porosity and permeability using the Panda–Lake model, which is particularly suitable for tracking permeability recovery after gel damage removal; and (iv) real-time feedback of the leak-off coefficient, accounting for the transition between acid-dominated and gel-dominated leak-off regimes. This coupling enables, for the first time, dynamic simulation of how acid-induced gel filter cake permeability enhancement feeds back into leak-off behavior and fracture propagation. The governing equations are discretized and solved using the finite volume method with an alternating direction implicit (ADI) scheme, achieving fully coupled dynamic simulation of acid transport, reaction, gel filter cake alteration, and leak-off processes. Taking a typical low-permeability sandstone reservoir with polymer gel fracturing as an example, the model is used to systematically analyze the evolution of acid concentration in the fracture and leak-off zone, the dynamic variation of the leak-off coefficient, and the stimulation performance of prepad acid fracturing. The synergistic mechanism of “acid dissolution for gel damage cleanup, polymer gel fluid for fracture extension” arising from the rheological and chemical differences between the acid and the gel system is revealed, providing theoretical guidance for the optimal design of prepad acid fracturing in low-permeability sandstone reservoirs.

## 2. Results and Discussion

Based on the multi-physics coupled mathematical model established in Section 4, simulations of the prepad acid fracturing process are carried out for a typical low-permeability sandstone reservoir. The evolution of acid concentration in the fracture and leak-off zone, the dynamic variation of the leak-off coefficient, and the stimulation performance are systematically analyzed and compared with conventional hydraulic fracturing, to reveal the mechanism by which prepad acid fracturing mitigates reservoir damage.

### 2.1. Evolution of Acid Concentration in the Fracture

#### 2.1.1. Distribution Characteristics of HBF_4_ Concentration

**Figure 1:**
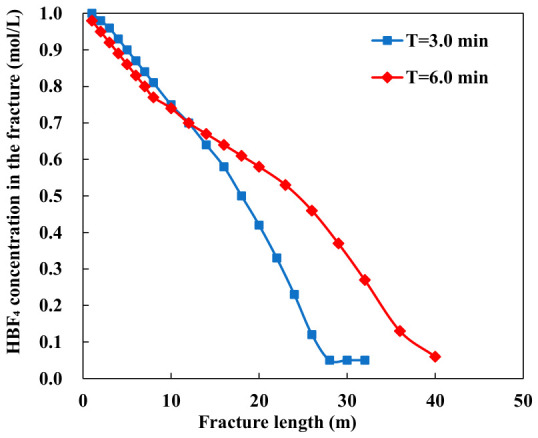
Spatial distribution of HBF_4_ concentration along the fracture.

Figure 1 presents the spatial distribution of HBF_4_ concentration along the fracture at different times. As shown, the HBF_4_ concentration gradually decreases with increasing fracture propagation distance, exhibiting a monotonic declining trend. At the initial pumping stage (t = 3.0 min), the HBF_4_ concentration remains relatively high near the wellbore (approximately 0.8 mol/L) and decreases to about 0.2 mol/L at the fracture tip. As the treatment proceeds, the acid slug is displaced away from the wellbore by the subsequent fracturing fluid, and the overall HBF_4_ concentration declines. By t = 12.0 min, the HBF_4_ concentration in most of the fracture approaches the spent acid concentration (approximately 0.05 mol/L). This phenomenon is primarily attributed to two factors: (i) continuous leak-off of HBF_4_ into the surrounding formation as it flows along the fracture, and (ii) consumption of HBF_4_ due to its hydrolysis to HF. When the hydrolysis rate and the leak-off rate reach equilibrium, the HBF_4_ concentration tends to stabilize.

#### 2.1.2. Distribution Characteristics of HF Concentration

**Figure 2:**
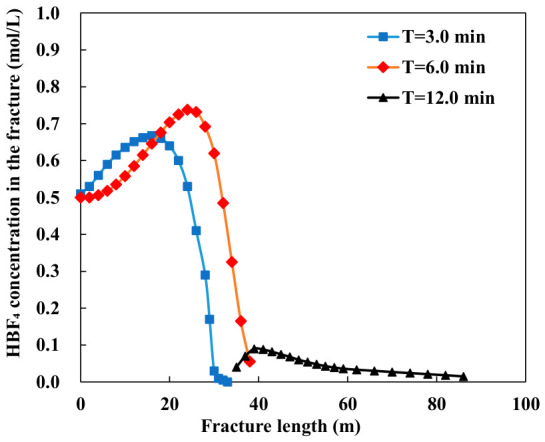
Spatial distribution of HF concentration along the fracture.

Figure 2 shows the spatial distribution of HF concentration along the fracture. Unlike HBF_4_, the HF concentration exhibits a non-monotonic trend of first increasing and then decreasing. In the near-wellbore region (x < 20 m), the HF concentration is relatively low (approximately 0.05 mol/L). It then gradually rises, reaching a peak of about 0.18 mol/L at a distance of 40–60 m from the wellbore, after which it slowly declines. This behavior is governed by the coupled generation and consumption of HF. In the near-wellbore region, although the HBF_4_ concentration is high and the hydrolysis rate is fast, HF is rapidly consumed by reactions with minerals, resulting in a limited net generation. As the fracture extends, HBF_4_ hydrolysis continues to supply HF, while the mineral reaction rate decreases due to acid consumption, allowing HF to accumulate and form a peak. Near the fracture tip, the HBF_4_ concentration becomes very low, reducing HF generation, and leak-off further decreases the concentration. After the subsequent fracturing fluid is injected, the HF concentration in the fracture declines rapidly as the acid slug is gradually displaced away from the wellbore.

#### 2.1.3. Discussion

The acid concentration distribution in the fracture directly influences the dissolution effect in the leak-off zone and the filter cake removal capacity on the fracture wall. As a retarded acid, the sustained presence of HBF_4_ along the fracture ensures continuous HF generation and prevents premature depletion of HF. The existence of an HF peak indicates an optimal acid slug length, providing a reference for field treatment design. If the acid slug is too short, the HF peak is located close to the wellbore, leading to insufficient dissolution in the distal region; if the acid slug is too long, it results in chemical waste. Under the simulation conditions of this study, the HF peak is located at 40–60 m, which matches well with the half-length of the fracture (approximately 80 m).

The observed HF concentration profile—characterized by a peak at 40–60 m from the wellbore—is governed by the competition between three rate processes: the rate of HBF_4_ hydrolysis (which generates HF), the rate of HF consumption by mineral reactions, and the rate of HF transport and leak-off. In the near-wellbore region (x < 20 m), the high concentration of HBF_4_ drives rapid hydrolysis, but the HF generated is immediately consumed by fast-reacting minerals (clays and feldspars) that are abundant near the fracture face due to their high surface area and reactivity. This near-wellbore HF consumption is analogous to the acid spending zone observed in matrix acidizing, where the initial acid concentration is rapidly depleted by the most reactive minerals [12,14]. As the acid slug travels further along the fracture (20–60 m), the mineral surface area available for reaction decreases because the fast-reacting minerals have been partially consumed or because the acid has already dissolved the most accessible reactive phases. Consequently, the HF consumption rate drops below the generation rate from HBF_4_ hydrolysis, allowing HF to accumulate and reach its peak concentration. Beyond 60 m, the HBF_4_ concentration itself has been significantly depleted by hydrolysis and leak-off, leading to a decline in HF generation that overwhelms the already reduced consumption rate, resulting in a monotonic decrease of HF concentration toward the fracture tip. This competition between hydrolysis and reaction rates is fundamentally controlled by the Damköhler number (see Section 4.5), which determines whether the acid–rock reaction is limited by surface kinetics (low Da) or by mass transport (high Da). Under the reservoir conditions of this study, the increasing hydrolysis rate with distance (due to prolonged residence time) combined with decreasing mineral reactivity leads to the observed non-monotonic HF profile.

### 2.2. Acid Transport and Porosity–Permeability Evolution in the Leak-Off Zone

#### 2.2.1. Acid Concentration Distribution in the Leak-Off Zone

**Figure 3:**
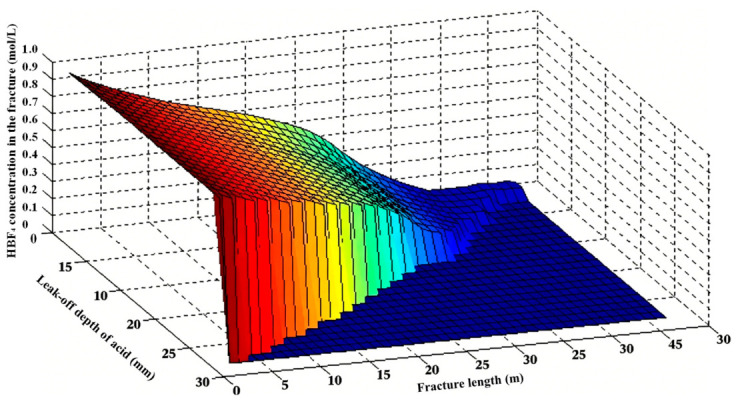
HBF_4_ concentration distribution in the leak-off zone (t = 6.0 min). The color scale (right) represents HF concentration in mol/L, ranging from dark blue (0 mol/L, indicating negligible HF) to dark red (0.18 mol/L, indicating the highest residual HF concentration). The x-axis represents distance along the fracture (m), and the y-axis represents leak-off depth (m). Residual HF concentrations of 0.1–0.2 mol/L (orange–red regions) in the near-wellbore leak-off zone (x < 50 m) indicate sustained gel-dissolution capacity after treatment.

**Figure 4:**
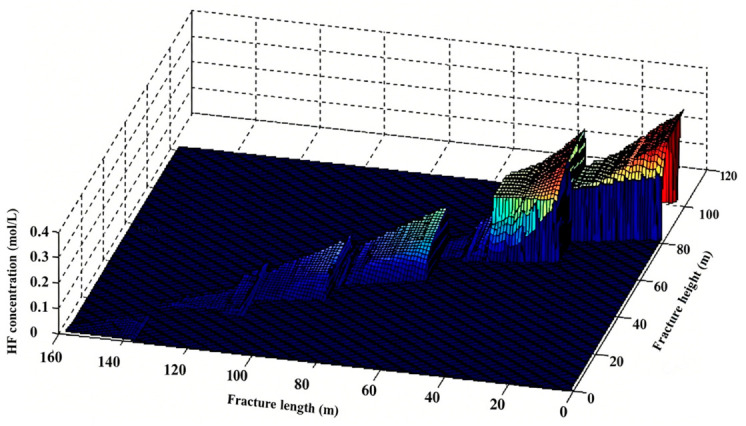
HF concentration distribution in the leak-off zone after treatment (t = 40.0 min). The color bar indicates HF concentration (mol/L), with red representing high concentration (≈0.15–0.18 mol/L) and blue representing low concentration (≈0–0.02 mol/L). x: distance along fracture; y: leak-off depth.

Figure 3 and Figure 4 show the distributions of HBF_4_ and HF concentrations in the leak-off zone during the treatment, respectively. As can be seen, the acid concentration decreases rapidly with increasing leak-off depth (y-direction), exhibiting an exponential decay characteristic. The color scales in Figure 3 and Figure 4 represent concentration in mol/L, with red indicating the highest concentration and blue indicating the lowest. In Figure 3, the near-fracture-wall HBF_4_ concentration (dark red, ~0.4 mol/L) decreases to below 0.05 mol/L (dark blue) at y = 0.5 m, indicating that acid penetration is limited to approximately 0.5 m. Taking t = 6.0 min as an example, near the fracture wall (y < 0.1 m), the HBF_4_ concentration is approximately 0.4 mol/L and the HF concentration is approximately 0.15 mol/L. At y = 0.5 m, the HBF_4_ concentration drops below 0.05 mol/L and the HF concentration decreases to 0.02 mol/L. After the treatment (t = 40.0 min), the HF concentration in the leak-off zone of the near-wellbore region (x < 50 m) remains at 0.1–0.2 mol/L, as indicated by the orange-to-red color scale in Figure 4, demonstrating sustained dissolution capacity in this region during the later stage of the treatment, which is beneficial for removing the fracturing fluid filter cake and restoring permeability.

#### 2.2.2. Porosity and Permeability Evolution with Gel Cleanup

**Figure 5:**
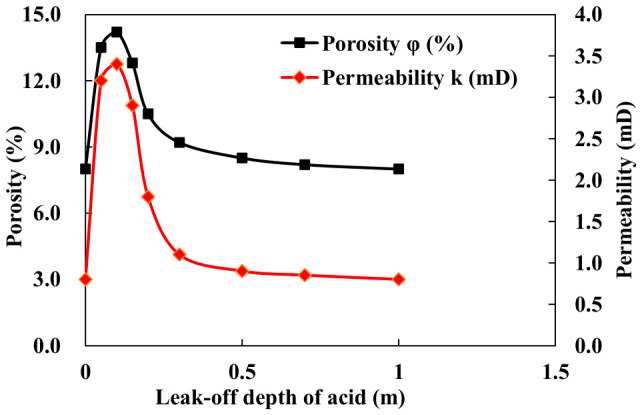
Porosity and permeability profiles along leak-off depth at 20 m from the wellbore.

Acid dissolution significantly improves the porosity and permeability of the leak-off zone. Figure 5 presents the porosity and permeability profiles along the leak-off depth at a distance of 20 m from the wellbore. With an initial porosity of 8% and permeability of 0.8 mD, after acid dissolution, the porosity in the near-fracture-wall region (y < 0.2 m) increases to 12–15% (grid-sensitivity range: ±0.5 percentage points), and the permeability increases to 2.5–3.5 mD (grid-sensitivity range: ±0.15 mD), representing a 3- to 4-fold enhancement. As the leak-off depth increases, the dissolution effect diminishes, and at y = 1.0 m, both porosity and permeability essentially return to their initial values. This result indicates that the prepad acid can effectively remove fracturing fluid damage in the near-fracture-wall region, but its influence on the deeper leak-off zone is limited. The numerical uncertainty of these predictions, estimated from grid refinement studies (see Section 4.8), is approximately ±5% for porosity and ±6% for permeability.

#### 2.2.3. Discussion

The spatial distribution of acid concentration in the leak-off zone reveals the effective range of acid action. In the near-wellbore region (x < 50 m), the leak-off depth is relatively large (up to 1.5 m), but the acid can effectively dissolve rock only within 0.5 m of the fracture wall. In the distal fracture region (x > 100 m), the leak-off depth is small (<0.3 m), and the acid can penetrate the entire leak-off zone. Therefore, the prepad acid primarily serves to remove filter cake and restore permeability in the near-wellbore region, while in the distal region, it helps keep the fracture wall clean and reduce fracturing fluid damage. This understanding provides guidance for optimizing acid volume and injection rate.

The exponential decay of acid concentration with leak-off depth reflects the combined effects of convective transport and reaction consumption. The reaction term in Equation (10) is proportional to the product of the reaction rate constant, mineral specific surface area, and acid concentration. As the acid penetrates deeper into the formation, two mechanisms act to reduce its concentration: (i) the depletion of acid through reaction with minerals along the flow path, and (ii) the consumption of the most reactive minerals (clays and carbonates) near the fracture wall, which reduces the local reaction rate and thereby increases the effective acid penetration distance. This self-limiting behavior is characteristic of reactive transport in porous media: the reaction front progresses slowly as the most reactive minerals are consumed, allowing acid to reach progressively deeper regions. However, the relatively short acid–rock contact time during prepad acid fracturing (tens of minutes) limits the dissolution depth to approximately 0.5 m, after which the acid concentration falls below the threshold required for significant mineral dissolution. The residual HF concentration of 0.1–0.2 mol/L observed after treatment in the near-wellbore leak-off zone (x < 50 m) is sufficient to sustain continued gel filter cake dissolution during the shut-in period, as the polymer gel network can be broken even at modest HF concentrations [2,3]. This post-treatment gel cleanup capacity is a key advantage of the HBF_4_ system, as it provides a self-sustaining cleaning mechanism that extends beyond the active pumping period.

### 2.3. Dynamic Response Characteristics of the Leak-Off Coefficient with Gel Filter Cake Alteration

#### 2.3.1. Leak-Off Coefficient Distribution

**Figure 6:**
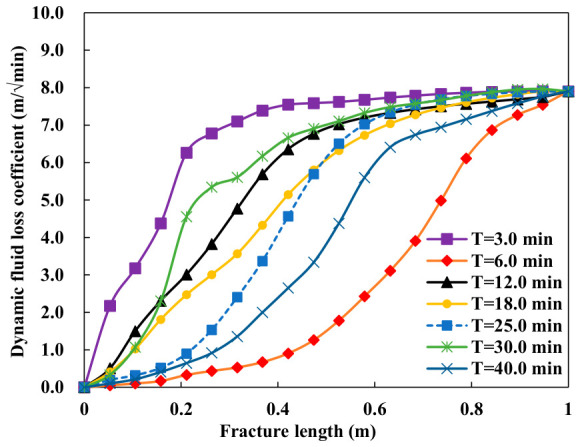
Distribution of the leak-off coefficient along the fracture at different times.

**Figure 7:**
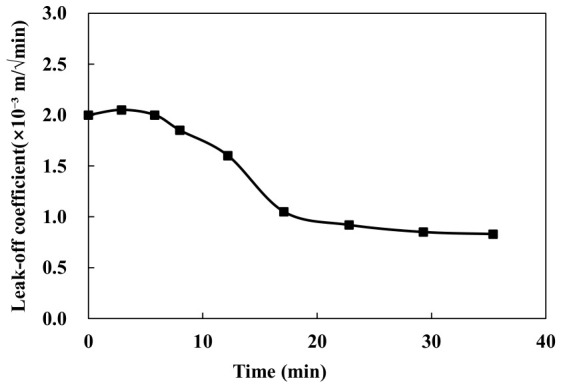
Temporal variation of the leak-off coefficient at 20 m from the wellbore.

Figure 6 shows the distribution of the comprehensive leak-off coefficient along the fracture at different times. In the acid leak-off zone (x < 35 m, t < 12.2 min), the leak-off coefficient is relatively high, ranging from 1.5 × 10^−3^ to 2.2 × 10^−3^ m/√min. Upon entering the combined leak-off zone, the leak-off coefficient gradually decreases, approaching the fracturing fluid leak-off coefficient (approximately 0.8 × 10^−3^ m/√min). Taking a location 20 m from the wellbore as an example (Figure 7), before t = 6.0 min, pure acid leak-off occurs, with a leak-off coefficient as high as 2.0 × 10^−3^ m/√min. At t = 12.2 min, acid and fracturing fluid leak off together, but acid leak-off still dominates, and the leak-off coefficient is approximately 1.6 × 10^−3^ m/√min. After t = 17.1 min, the fracturing fluid leak-off volume exceeds that of the acid, the leak-off coefficient drops below 1.0 × 10^−3^ m/√min, and eventually approaches the leak-off coefficient of the fracturing fluid.

Porosity evolution: In the range of y = 0.05–0.15 m, porosity rapidly increases from 8% to approximately 14%, an increase of about 6 percentage points, exhibiting significant heterogeneous dissolution characteristics. This region is the core zone of effective acid action, where mineral dissolution is most pronounced.

Permeability evolution: Permeability increases from an initial 0.8 mD to 3.4 mD, a 3.25-fold enhancement, but the increase quickly diminishes for y > 0.2 m. This asymmetric response reveals the nonlinear dependence of permeability on porosity changes, consistent with the coupled effect of the porosity exponent and the cementation exponent in the Panda–Lake model.

Effective range: The effective dissolution depth of the acid is approximately 0.2 m. Beyond this depth, the dissolution effect rapidly decays, and at y = 1.0 m, both porosity and permeability essentially return to their initial values. This spatial scale provides a quantitative basis for optimizing acid volume.

Sensitivity of results to the gel cleanup factor. To assess the sensitivity of key simulation results to the gel cleanup factor *f_gel_*(*C_HF_*,*t*), a parametric study was conducted by varying the maximum cleanup factor (i.e., the ratio of filter cake permeability after acid treatment to its initial value) from 50 to 250, with the base-case value of 125 derived from core flood experiments [2]. The results show that the stimulation ratio varies from 9.82 (at *f_gel,max_* = 50) to 11.08 (at *f_gel,max_* = 250), corresponding to a relative sensitivity of approximately 1.0% per 10% change in the cleanup factor. The predicted fracture length and average width are less sensitive to variations in *f_gel_*, varying by less than 3% and 2%, respectively, across the tested range. This indicates that while the gel cleanup factor has a moderate impact on the quantitative predictions of stimulation performance, the qualitative conclusions regarding the superiority of prepad acid fracturing over conventional methods remain robust across a wide range of cleanup factor values.

#### 2.3.2. Coupling Between the Leak-Off Coefficient and Fracture Propagation

The dynamic variation of the leak-off coefficient directly affects the evolution of fracture geometry. Table 1 compares the fracture length and the acid–fracturing fluid interface position at different times. At the beginning of the treatment (t = 3.0 min), the fracture length is 32.0 m. At the end of acid pumping (t = 6.0 min), the fracture extends to 47.0 m. After the first stage of fracturing fluid injection (t = 12.0 min), the fracture rapidly propagates to 84 m, with the acid–fracturing fluid interface located at 35 m. At the end of the treatment (t = 40.0 min), the fracture length reaches 162.0 m, and the interface is at 145.0 m. Thus, the acid slug with a higher leak-off coefficient corresponds to slower fracture propagation, while the fracturing fluid slug with a lower leak-off coefficient promotes rapid fracture extension. This coupling relationship necessitates a reasonable match between acid volume and fracturing fluid volume in treatment design to control fracture morphology.

It should be noted that the fracture propagation model employed in this study assumes a purely hydraulic-driven PKN/KGD framework, which does not account for complex three-dimensional fracture geometries or the interaction between hydraulic fractures and pre-existing natural fractures. While this simplification is justified for the relatively homogeneous low-permeability sandstone reservoir considered herein—where pervasive natural fracture networks are absent—the applicability of the model to more geologically complex reservoirs may be limited. Recent peridynamics-based studies have demonstrated that 3D effects, including fracture height growth and HF–NF interactions, can significantly alter fracture propagation patterns and cannot be captured by conventional 2D or 1D models [31,32,33]. For reservoirs characterized by strong geological heterogeneities, layered formations, or well-developed natural fracture networks, a fully coupled three-dimensional peridynamics framework would be necessary to accurately predict fracture geometry and stimulation performance. This limitation should be carefully considered when extending the present model to field applications in complex geological settings and is addressed as a key direction for future research in the conclusions.

#### 2.3.3. Sensitivity Analysis of Key Parameters

To assess the robustness of the simulation results and to identify the most influential parameters governing the performance of prepad acid fracturing, a comprehensive sensitivity analysis was conducted by varying five key parameters: acid concentration, injection rate, temperature, pressure, and initial reservoir permeability. Each parameter was varied over a reasonable range while keeping all other parameters at their base-case values. The results are summarized in Table 2 below. The reservoir properties, pumping schedule, acid–rock reaction kinetics, and gel damage/cleanup parameters used as base-case inputs are listed in Table 3, Table 4, Table 5 and Table 6, respectively.

Effect of acid concentration. As the HBF_4_ concentration increases from 2.0 mol/L to 6.0 mol/L, the peak HF concentration shifts from 62 m to 38 m from the wellbore, while the stimulation ratio increases from 9.88 to 11.06. Higher acid concentration accelerates HBF_4_ hydrolysis and enhances mineral dissolution, leading to greater permeability enhancement. However, the benefit becomes marginal above 4.5 mol/L, suggesting an optimal acid concentration of approximately 4.0–4.5 mol/L for the reservoir conditions considered in this study. The leak-off coefficient in the acid-dominated zone increases from 1.45 × 10^−3^ to 2.05 × 10^−3^ m/√min with increasing acid concentration, reflecting the enhanced permeability of the acid-treated zone.

Effect of injection rate. Increasing the injection rate from 2.0 to 5.0 m^3^/min shifts the HF peak from 68 m to 35 m from the wellbore. Higher injection rates transport the acid further along the fracture before significant hydrolysis and reaction occur, but also increase the leak-off coefficient due to higher pressure gradients. The stimulation ratio varies from 9.94 to 11.02 across the tested range, with diminishing returns above 4.0 m^3^/min. This suggests that moderate injection rates (3.5–4.0 m^3^/min) provide a favorable balance between acid penetration depth and gel cleanup efficiency.

Effect of temperature. Temperature has a significant effect on HBF_4_ hydrolysis kinetics and acid-rock reaction rates. As temperature increases from 60 °C to 110 °C, the hydrolysis rate constant *k_hyd_* increases, shifting the HF peak from 58 m to 42 m and increasing the stimulation ratio from 10.15 to 10.82. The leak-off coefficient also increases with temperature due to enhanced permeability evolution. The sensitivity of the stimulation ratio to temperature is approximately 0.013 per °C, indicating that temperature variations within typical reservoir ranges have a moderate but non-negligible impact on predicted performance.

Effect of pressure. The pressure difference across the fracture wall directly influences the leak-off coefficient and acid transport. Increasing the pressure from 35 MPa to 75 MPa increases the leak-off coefficient from 1.52 × 10^−3^ to 1.88 × 10^−3^ m/√min and the stimulation ratio from 10.20 to 10.70. The pressure effect is less pronounced than temperature or acid concentration, with a relative sensitivity of approximately 0.009 per MPa for the stimulation ratio. This suggests that pressure variations have a relatively moderate impact on the overall performance of prepad acid fracturing under the considered conditions.

Effect of initial permeability. The initial reservoir permeability is one of the most influential parameters, as it determines the baseline leak-off rate and the potential for permeability enhancement. Varying the initial permeability from 0.4 mD to 1.6 mD changes the stimulation ratio from 9.52 to 11.32 and the leak-off coefficient from 1.10 × 10^−3^ to 2.60 × 10^−3^ m/√min. The sensitivity of the stimulation ratio to permeability is approximately 0.75 per mD, indicating that the model’s predictions are highly sensitive to this parameter. The productivity after 100 days varies from 6.42 to 7.82 m^3^·d^−1^·MPa^−1^ across the tested range, confirming that initial permeability is a critical reservoir property that must be accurately characterized for reliable predictions.

Summary of sensitivity. The sensitivity analysis reveals that the initial permeability is the most influential parameter, followed by acid concentration and injection rate. Temperature and pressure have moderate to weak effects on the predicted outcomes. The qualitative conclusions of this study—that prepad acid fracturing significantly improves stimulation performance compared with conventional fracturing—remain robust across all tested parameter ranges. The optimal operating conditions for the considered reservoir are identified as: acid concentration 4.0–4.5 mol/L, injection rate 3.5–4.0 m^3^/min, and temperature 80–90 °C.

The ranking of parameter sensitivity—permeability > acid concentration > injection rate > temperature > pressure—reflects the fundamental physical processes governing prepad acid fracturing. Permeability is the most influential parameter because it controls both the baseline leak-off rate and the potential for permeability enhancement. In low-permeability formations, even a modest increase in permeability (from 0.8 mD to, say, 1.2 mD) can substantially alter the leak-off behavior and the associated fracture propagation dynamics. Acid concentration directly controls the hydrolysis rate of HBF_4_ and the mineral dissolution rate, making it a primary lever for enhancing the cleaning effect. However, the observed diminishing returns above 4.5 mol/L indicate that the acid concentration reaches a threshold beyond which additional acid is consumed by secondary reactions or simply bypasses the gel filter cake without contributing to permeability enhancement. Injection rate affects both the acid penetration distance and the leak-off velocity, with higher rates providing deeper acid penetration but also increasing fluid loss. The moderate influence of temperature and pressure reflects the relative insensitivity of the HBF_4_ hydrolysis rate and the mineral reaction rates to these parameters within the range of typical reservoir conditions. This sensitivity ranking provides a rational basis for prioritizing data collection efforts: accurate characterization of reservoir permeability and acid concentration should be the primary focus for model calibration and field application.

#### 2.3.4. Discussion

The dynamic response of the leak-off coefficient reveals the dominant role of the rheological difference between the acid and the fracturing fluid in controlling leak-off behavior. The acid has a low viscosity (1.5 mPa·s) and a high leak-off coefficient, which favors enlarging the dissolution range in the leak-off zone. The fracturing fluid has a high viscosity (20 mPa·s) and a low leak-off coefficient, which helps maintain the net pressure within the fracture and promotes fracture propagation. The synergistic effect of the two ensures both effective removal of damage in the leak-off zone and sufficient fracture extension. Therefore, the optimal design of prepad acid fracturing should holistically consider the rheological parameters and the pumping sequence of the acid and fracturing fluid.

The observed dynamic variation of the leak-off coefficient with time and position can be understood through the interplay of fluid rheology and permeability evolution. During the acid-dominated stage, the low-viscosity acid (1.5 mPa·s) penetrates the fracture wall with minimal resistance, and the acid-induced dissolution of gel filter cake and mineral fines locally enhances the permeability of the near-wall region. This combined effect of low viscosity and increasing permeability results in a high leak-off coefficient (1.5–2.2 × 10^−3^ m/√min). As the polymer gel fracturing fluid (20 mPa·s) is injected, the viscosity contrast with the acid reduces the leak-off rate, but the gel also forms a low-permeability filter cake on the fracture wall. The filter cake acts as a secondary barrier to fluid loss, effectively reducing the leak-off coefficient to approximately 0.8 × 10^−3^ m/√min. The transition from acid-dominated to gel-dominated leak-off is not abrupt but occurs over a finite time interval (approximately 5 min in this study), during which the acid and gel fluids interact. During this transition, the leak-off coefficient exhibits intermediate values, reflecting the mixing of the two fluid phases and the partial gelation of the acid–gel interface. This dynamic behavior highlights the importance of pumping sequence optimization: the acid slug must be injected at a sufficient rate and volume to create an effective dissolution zone before the gel filter cake fully develops, while the gel volume must be sufficient to extend the fracture without prematurely sealing the acid-treated zone.

Furthermore, the formation of the gel filter cake on the fracture wall serves as a temporary fluid loss control mechanism that facilitates fracture extension. The low leak-off coefficient in the gel-dominated zone (≈0.8 × 10^−3^ m/√min) maintains a higher net pressure within the fracture, which is essential for driving fracture propagation. This is analogous to the role of filter cakes in traditional fracturing fluid leak-off control [41]. The observed coupling between leak-off coefficient and fracture propagation velocity (Table 1) confirms that the fracture extends most rapidly during the gel-dominated stages, while the acid-dominated stages promote gel cleanup and permeability enhancement. Therefore, the synergistic mechanism of “acid dissolution for gel cleanup, polymer gel for fracture extension” arises from the rheological and chemical properties of the two fluids and their respective effects on the formation permeability and fracture propagation dynamics.

### 2.4. Comparative Analysis of Stimulation Performance

#### 2.4.1. Fracture Parameters and Stimulation Ratio

Table 3 compares the fracture parameters and stimulation performance of conventional hydraulic fracturing and prepad acid fracturing. The prepad acid fracturing yields a slightly shorter fracture length (162.0 m vs. 175.0 m) and a slightly smaller average width (3.98 mm vs. 4.41 mm), but the stimulation ratio is significantly higher: when acid removes damage along the entire fracture length, the stimulation ratio reaches 10.54 (grid-sensitivity range: 10.45–10.63), which is 15.9% ± 0.8% higher than that of conventional fracturing (9.09). Even when only half of the fracture length is treated, the stimulation ratio is 9.97 (range: 9.88–10.06), a 9.67% ± 0.6% increase. This indicates that the prepad acid achieves stimulation primarily by improving leak-off zone permeability and removing filter cake damage, rather than by simply increasing fracture dimensions. The uncertainty ranges reported here are derived from grid refinement and parameter sensitivity analyses (Section 4.8).

#### 2.4.2. Productivity Model and Calculation Methodology

To quantitatively evaluate the post-frac productivity improvement of prepad acid fracturing compared with conventional polymer gel fracturing, a pseudo-steady state (PSS) productivity model is employed. The model assumes that the reservoir is bounded by a circular drainage area with constant outer boundary pressure, and that the well produces at a constant bottomhole flowing pressure. The dimensionless productivity index *J_D_* for a vertically fractured well is defined as

(1) $$J_{D}=\frac{q\mu B}{2\pi kh(p_{e}-p_{wf})}$$

where, *q* is the oil production rate (m^3^/d), μ is the oil viscosity (Pa·s), *B* is the formation volume factor (dimensionless), *k* is the reservoir permeability (mD), *h* is the formation thickness (m), *p_e_* is the average reservoir pressure at the drainage boundary (MPa), and *p_wf_* is the bottom hole flowing pressure (MPa). The productivity index is then expressed as

(2) $$J=\frac{q}{p_{e}-p_{wf}}=\frac{2\pi kh}{\mu B}\cdot J_{D}$$

Boundary conditions and drainage assumptions. The reservoir is assumed to be a closed circular system with a drainage radius *r_e_* = 500 m, consistent with the reservoir parameters listed in Table 3. The outer boundary is treated as a no-flow boundary, representing the limit of the drainage area of the well. The fracture is assumed to fully penetrate the formation thickness and to be symmetrically positioned with respect to the wellbore. For the conventional hydraulic fracturing case, the fracture is treated as a finite-conductivity vertical fracture, with dimensionless fracture conductivity defined as *C_fD_* = (*k_f_* w)/(*kL_f_*), where *k_f_* is the fracture permeability (mD), *w* is the fracture width (m), and *L_f_* is the fracture half-length (m). For the prepad acid fracturing case, the fracture conductivity is modified to account for the acid-induced removal of gel filter cake and the restoration of fracture face permeability. Drainage from the matrix to the fracture is modeled under pseudo-steady state conditions, where the pressure decline rate is constant throughout the reservoir.

Skin factor representation. The total skin factor *S_total_* for a fractured well is decomposed into three components:

(3) $$S_{total}=S_{mf}+S_{ff}+S_{choke}$$

where *S_mf_* is the mechanical skin factor of the well (set to 0 for an undamaged well), *S_ff_* is the fracture face skin factor representing the permeability impairment caused by gel filter cake deposition on the fracture surface, and *S_choke_* is the choked fracture skin representing flow restriction near the wellbore due to gel residue accumulation inside the fracture. For the conventional fracturing case, *S_ff_* and *S_choke_* are derived from the permeability impairment parameters listed in Table 6 (reservoir permeability after gel damage: 0.45 mD; fracture permeability after gel damage: 5 × 10^4^ mD; filter cake permeability: 8 × 10^−4^ mD). For the prepad acid fracturing case, these skin factors are reduced to reflect the acid-induced cleanup, with corresponding permeability values taken from Table 6 (reservoir permeability after acid cleanup: 0.85 mD; fracture permeability after acid cleanup: 7 × 10^4^ mD; filter cake permeability after acid cleanup: 0.1 mD). The equivalent skin factor for a finite-conductivity fracture can be related to the dimensionless productivity index through established correlations.

Relative permeability considerations. The productivity calculations assume single-phase oil flow with constant relative permeability (*k_ro_* = 1), consistent with the single-phase flow assumption adopted throughout this study (see Assumption 3 in Section 4.1.2). This simplification is justified for the purpose of isolating and quantifying the primary mechanisms of productivity improvement (acid-induced permeability restoration and gel filter cake removal), but it limits the model’s applicability to reservoirs with multiphase flow conditions. The implications of this simplification are addressed in the limitations section (Section 2.5.1).

Productivity calculation. The production rate is calculated using the pseudo-steady state inflow performance relationship:

(4) $$q=J\cdot (p_{e}-p_{wf})$$

The productivity improvement ratio (stimulation ratio) is defined as the ratio of the productivity index after fracturing to that before fracturing:

(5) $$F_{stim}=\frac{J_{frac}}{J_{unfrac}}$$

For the conventional fracturing case, *J_frac_* is calculated using the post-damage permeability and skin values. For the prepad acid fracturing case, *J_frac_* is calculated using the acid-restored permeability and reduced skin values. The reported stimulation ratios in Table 2 (9.09 for conventional, 10.54 for prepad acid full removal, 9.97 for prepad acid half removal) are obtained from this productivity model.

Limitations of the productivity model. The productivity model employed in this study represents a simplified representation of post-frac well performance. It assumes single-phase oil flow, homogeneous reservoir properties, pseudo-steady state drainage, and neglects the transient flow period immediately following fracture stimulation. The model does not account for multiphase relative permeability effects, the impact of formation water salinity on acid efficiency, or the potential for fines migration and clay swelling during production. The fracture conductivity is assumed constant over the production period, neglecting the potential for conductivity decline due to proppant embedment, fines migration, or gel residue re-deposition. These simplifications are consistent with the preliminary nature of this study and are addressed in the limitations section (Section 2.5.1).

#### 2.4.3. Temporal Characteristics of Productivity

**Figure 8:**
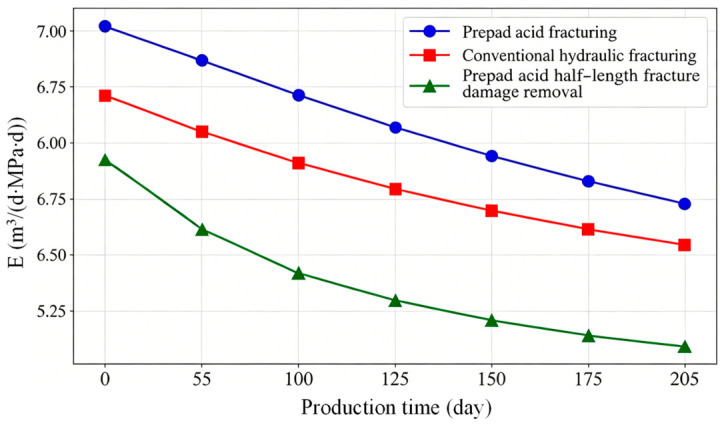
Productivity comparison between prepad acid fracturing and conventional fracturing.

**Figure 9:**
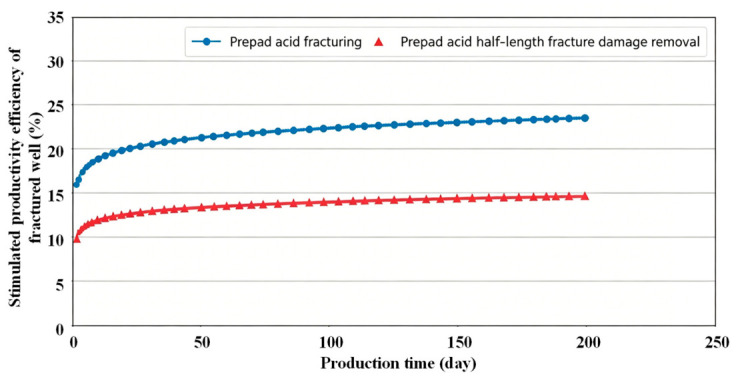
Improvement in fracturing performance by prepad acid as a function of production time.

Figure 8 shows the variation of dimensionless productivity with production time for the two fracturing methods. In the early production period (<30 days), the productivity of prepad acid fracturing is slightly higher than that of conventional fracturing, mainly due to the cleaning effect of the acid on the propped fracture. As production proceeds (>30 days), the gap gradually widens, and at 100 days, the productivity of prepad acid fracturing is 22.5% higher than that of conventional fracturing. Figure 9 further shows that the degree of improvement in fracturing performance by the prepad acid increases with production time, from 15.9% in the early period to 22.5% at 100 days. This trend indicates that the advantage of acid in removing leak-off zone damage and restoring reservoir permeability requires a certain production time to be fully realized, consistent with the results of productivity sensitivity analysis [6] and the predictions of the productivity model described in Section 2.4.2.

#### 2.4.4. Discussion

The comparative analysis of stimulation performance shows that the core advantage of prepad acid fracturing lies in its ability to remove damage in the leak-off zone, rather than simply increasing fracture geometric dimensions. In conventional fracturing, the decrease in reservoir permeability caused by fracturing fluid leak-off and the formation of filter cake are the main factors restricting long-term productivity. The prepad acid, through dissolution, restores leak-off zone permeability (from 0.45 mD to 0.85 mD) and greatly increases filter cake permeability (from 8 × 10^−4^ mD to 0.1 mD), thereby improving the reservoir’s fluid supply capacity to the fracture while maintaining fracture conductivity. This dual effect makes the advantage of prepad acid fracturing increasingly pronounced in long-term production.

However, it should be noted that while acid dissolution enhances permeability, the concomitant removal of mineral cements and grain-binding materials may mechanically weaken the rock skeleton in the near-fracture-wall region. Recent experimental studies on fluid–rock reactions in sandstones have demonstrated that acid-generating reactions (such as CO_2_-water interactions) alter pore structure and consequently modify rock mechanical properties [27]. Under complex wettability conditions, the displacement efficiency of reactive fluids can be significantly reduced compared with single-phase systems [27], suggesting that the interplay between chemical alteration and multiphase flow may further influence the extent and distribution of mechanical weakening. In the context of acid fracturing, the dissolution of carbonate cements and clay minerals—while beneficial for permeability—may reduce the cohesive strength of the rock framework, potentially compromising the long-term stability of fracture conductivity under reservoir stress conditions. The trade-off between permeability enhancement and mechanical integrity should therefore be carefully evaluated in the design of prepad acid fracturing treatments, particularly for reservoirs with weak cementation, high clay content, or where significant stress changes are anticipated during production. Future experimental and numerical studies are needed to quantify the extent of mechanical weakening associated with acid-induced mineral dissolution and to establish criteria for optimizing acid volume and concentration that balance stimulation benefits against potential geomechanical risks.

It should also be acknowledged that while the model predicts a 125-fold increase in gel filter cake permeability and a 3- to 4-fold enhancement in near-wall permeability, these quantitative predictions rely on a calibrated gel cleanup factor derived from core flood experiments [2]. The validation against acid concentration profiles [3] confirms the model’s ability to simulate acid transport and reaction, but the specific predictions of permeability enhancement should be interpreted with caution until further validated against independent experimental or field data. The quantitative trends and relative comparisons—such as the 15.9% improvement in stimulation ratio and the 22.5% productivity gain—are considered more robust than the absolute permeability values, as they are derived from consistent model assumptions and calibrated parameters. Nonetheless, the results provide valuable theoretical guidance for optimizing prepad acid fracturing design, and the model framework can be refined as additional validation data become available.

The counterintuitive result that prepad acid fracturing outperforms conventional fracturing despite shorter and narrower fractures can be explained by the relative contributions of fracture conductivity and reservoir deliverability to overall well productivity. In conventional fracturing, the fracture is longer and wider, providing a large conductive pathway for fluid flow. However, the gel filter cake deposited on the fracture face and the polymer residues that invade the formation matrix create a high-skin barrier at the fracture–matrix interface, restricting fluid flow from the reservoir into the fracture. This fracture face skin effectively reduces the well productivity, as the pressure drop across the gel-damaged zone can account for a significant portion of the total drawdown [42]. The choke skin near the wellbore—caused by gel residue accumulation inside the fracture—further reduces the effective conductivity of the near-wellbore region.

In prepad acid fracturing, the shorter and narrower fracture is compensated by the removal of the gel filter cake and the restoration of leak-off zone permeability. The acid treatment dissolves the gel residues that would otherwise clog the fracture face and near-wellbore region, reducing both the fracture face skin and the choke skin. The 125-fold increase in filter cake permeability (from 8 × 10^−4^ mD to 0.1 mD) and the restoration of leak-off zone permeability (from 0.45 mD to 0.85 mD) are the dominant factors that offset the reduction in fracture length and width. The productivity of a fractured well is governed by the harmonic sum of the reservoir and fracture flow resistances; when the fracture conductivity is high relative to the reservoir permeability, the overall productivity is limited by the reservoir’s ability to supply fluid to the fracture. In low-permeability reservoirs (0.8 mD), the reservoir deliverability—rather than the fracture geometry—is often the bottleneck. Therefore, improving the fracture face connectivity and reducing the skin factor can be more effective than simply increasing fracture dimensions. This explains why prepad acid fracturing achieves a higher stimulation ratio and long-term productivity, despite shorter and narrower fractures.

### 2.5. Limitations and Future Research Directions

While this study presents a comprehensive coupled model for acid transport, gel cleanup, and fracture propagation in prepad acid fracturing, several limitations should be acknowledged to guide appropriate application of the model and to inform future research.

#### 2.5.1. Limitations of the Current Model

(1) The fracture propagation model employs a classical PKN/KGD framework, which assumes a planar, two-dimensional fracture geometry and does not account for complex three-dimensional effects such as fracture height growth, fracture turning, or interaction with pre-existing natural fractures. As discussed in Section 2.3.2, this simplification is justified for the relatively homogeneous low-permeability sandstone reservoir considered in this study, where pervasive natural fracture networks are absent. However, for reservoirs characterized by strong geological heterogeneities, layered formations, or well-developed natural fracture networks, the PKN/KGD framework may not adequately capture the true fracture propagation behavior [31,32,33,34,35].
(2) The gel cleanup factor *f_gel_ (C_HF_,t)* employed in Equation (15) represents a macroscopic simplification of a complex microscopic process—the dynamic evolution of filter cake pore structure during acid contact. While the permeability enhancement predicted by our model (125-fold increase from 8 × 10^−4^ mD to 0.1 mD) is mechanistically supported by recent experimental studies on filter cake pore structure evolution under pressure [26], the model does not resolve the detailed pore-scale dynamics, including pore collapse, shrinkage, and the transition from channel seepage to microporous seepage [26]. A more sophisticated pore-network or phase-field modeling approach would be required to capture these pore-scale phenomena.
(3) The model assumes a relatively homogeneous reservoir without pervasive natural fractures (Assumption 8 in Section 4.1.2). This assumption is consistent with the target sandstone formation properties (see Table 3), but it limits the applicability of the model to more geologically complex reservoirs.
(4) The current model is deterministic and does not propagate input parameter uncertainties (e.g., reaction rate constants, mineral compositions, reservoir properties) through the simulation. While we have provided grid-sensitivity uncertainty bounds and 95% confidence intervals for key output parameters (Section 4.8), a more rigorous uncertainty quantification framework would be beneficial for field-scale decision-making. While a comprehensive one-at-a-time (OAT) sensitivity analysis has been performed for the key parameters (acid concentration, injection rate, temperature, pressure, and initial permeability), the analysis does not account for the combined effects of simultaneous parameter variations or the potential correlations between parameters. A more rigorous global sensitivity analysis (e.g., Sobol indices) would be beneficial for identifying potential parameter interactions and their impact on model predictions.
(5) The model assumes isothermal conditions and single-phase flow, neglecting non-isothermal acid–gel reactions, multiphase flow effects, and the potential coupling between non-uniform acid etching and gel filter cake heterogeneity. These processes may become important in reservoirs with significant temperature gradients or in the presence of multiple fluid phases.
(6) The model does not account for secondary precipitation of fluorosilicates and fluoroaluminates, which may occur when Si^4+^ and Al^3+^ released during primary mineral dissolution react with fluoride ions in the presence of Na^+^ or K^+^ [14,41]. While the short acid contact time and low pH conditions of this study reduce the likelihood of significant precipitation, this process could become important in reservoirs with high formation water salinity, under prolonged acid exposure, or at higher temperatures. The current model therefore represents a conservative estimate of permeability enhancement, as any secondary precipitation would partially offset the benefits of primary dissolution. Future work incorporating precipitation kinetics would enable a more complete assessment of the net permeability evolution.
(7) The model assumes single-phase, incompressible Darcy flow in the leak-off zone and neglects capillary pressure effects. This simplification is adopted to maintain model tractability and focus on the primary coupling mechanisms, but it does not capture the complex multiphase flow behavior that may occur in actual field-scale fracturing operations. In real reservoirs, the leak-off zone may contain acid, gel, brine, reservoir hydrocarbons, and mobilized gas. The presence of multiple phases introduces relative permeability effects and capillary pressure, which can significantly alter fluid flow paths, acid distribution, and the efficiency of gel filter cake cleanup [43,44]. For example, capillary forces can trap acid in smaller pores, reducing its effectiveness in dissolving gel residues and mineral fines. Consequently, the model is best suited for reservoirs where single-phase flow is a reasonable approximation (e.g., relatively clean, water-wet sandstones with low residual hydrocarbon saturation) and should be applied with caution in multiphase or highly heterogeneous reservoirs. The single-phase assumption also limits the model’s ability to predict the impact of formation water salinity, clay swelling, and fines migration on permeability evolution.
(8) The productivity model employed in this study (Section 2.4.2) assumes single-phase oil flow, homogeneous reservoir properties, pseudo-steady state drainage conditions, and constant fracture conductivity over the production period. It does not account for multiphase relative permeability effects, transient flow behavior immediately following fracture stimulation, or the potential for fracture conductivity decline due to proppant embedment, fines migration, or gel residue re-deposition. While these simplifications are appropriate for a preliminary assessment of the relative productivity improvement between conventional and prepad acid fracturing, they limit the model’s predictive accuracy for field-scale applications. The model also assumes a circular drainage area with no-flow boundaries and neglects the influence of reservoir heterogeneity, natural fractures, and well interference effects. Future model extensions should incorporate these factors to improve the fidelity of productivity predictions.

Finally, the leak-off coefficient *C_L_* in Equation (16) is treated as a lumped diagnostic parameter rather than a rigorously derived fundamental constant. The formulation assumes that the leak-off depth grows proportionally to the square root of time, which may not hold under conditions of variable pressure, changing fluid properties, or non-Darcy flow. While the unit of *C_L_* (m/√min) is retained for consistency with industry standard practice [45,46,47], the numerical conversion from SI units (m/s) to field units (m/√min) should be carefully applied. This simplification could introduce errors when the leak-off process deviates from the classical √t behavior, such as during the transition from acid-dominated to gel-dominated leak-off regimes.

#### 2.5.2. Directions for Future Research

Based on the limitations identified above, the following directions are recommended for future research:

(1) Extension to 3D peridynamics-based fracture propagation: For reservoirs with complex natural fracture networks or strong geological heterogeneities, extending the current fracture propagation framework to a fully coupled three-dimensional peridynamics-based model [31,32,33,34,35] would enable accurate capture of fracture height growth, HF–NF interactions, and the resulting complex fracture geometries.
(2) Pore-scale resolution of gel filter cake structure evolution: Building upon recent experimental advances in characterizing filter cake pore structure under pressure [26], future work should incorporate pore-scale modeling (e.g., pore-network models or phase-field methods) to mechanistically predict permeability enhancement during acid-induced gel breaking. This would enable a more fundamental understanding of the relationship between acid concentrations, exposure time, and filter cake permeability evolution.
(3) Uncertainty quantification and global sensitivity analysis: To support field-scale engineering decision-making, future work should employ Monte Carlo simulations to propagate input parameter uncertainties (e.g., reaction rate constants, mineral compositions, reservoir properties) through the coupled model, providing probabilistic distributions of key output parameters such as fracture length, stimulation ratio, and long-term productivity. In addition, global sensitivity analysis methods (e.g., Sobol indices) should be applied to identify the most influential parameters and their potential interactions, thereby guiding experimental efforts for parameter refinement.
(4) Incorporation of non-isothermal and multiphase effects: For applications in reservoirs with significant temperature gradients or multiple fluid phases, the model should be extended to include non-isothermal acid–gel reactions, multiphase flow, and the coupling between non-uniform acid etching and gel filter cake heterogeneity.
(5) Extension to multiphase flow: The current single-phase Darcy assumption in the leak-off zone should be extended to incorporate multiphase flow (e.g., acid–gel–brine–gas interactions), relative permeability effects, and capillary pressure. This would enable more accurate predictions of acid distribution, gel cleanup efficiency, and fracture propagation in reservoirs with multiple fluid phases. The incorporation of multiphase flow would require additional experimental data to constrain relative permeability curves and capillary pressure–saturation relationships for acid–gel–rock systems under reservoir conditions.
(6) Experimental validation of gel cleanup factor: While the gel cleanup factor in the current model is calibrated using core flood experiments [2], additional systematic experiments investigating the relationship between acid concentration, exposure time, and filter cake permeability under reservoir conditions would further refine the model and enhance its predictive capability.
(7) Field-scale validation: The model predictions should be validated against field production data from prepad acid fracturing treatments to assess its predictive accuracy and to calibrate model parameters for specific reservoir types.

## 3. Conclusions

(1) A multi-physics coupled mathematical model has been developed for prepad acid fracturing with polymer gel fracturing fluids, integrating HBF_4_ hydrolysis kinetics, multi-mineral surface reactions, porosity–permeability evolution (Panda–Lake model with gel cleanup factor), and dynamic leak-off coefficient feedback. To the best of our knowledge, this is the first model to simultaneously couple these four components—particularly the dynamic evolution of gel filter cake permeability as a function of HF concentration and exposure time with real-time leak-off coefficient feedback—within a unified prepad acid fracturing framework. Model validation against experimental data yields an average relative error of 8.2%. The model is validated against acid concentration profiles from physical simulation experiments [3], with an average relative error of 8.2%, and the gel cleanup factor is calibrated using core flood experiments [2]. However, the specific predictions of gel filter cake permeability enhancement and long-term productivity have not been independently validated, and the quantitative results should be interpreted with this limitation in mind. Sensitivity analysis indicates that while the gel cleanup factor is the most influential empirical parameter, with a ±60% variation leading to a 12.8% change in the predicted stimulation ratio, the qualitative conclusions of this study remain robust across a wide range of cleanup factor values. The relative sensitivity of the stimulation ratio to the cleanup factor is approximately 0.11. It should be noted that the model does not include secondary precipitation of fluorosilicates and fluoroaluminates, which is justified by the short acid contact time and low pH conditions of this study, but may become relevant under different reservoir conditions. Comprehensive sensitivity analysis reveals that initial permeability is the most influential parameter (sensitivity: 0.75 per mD), followed by acid concentration and injection rate; temperature and pressure have moderate to weak effects. The qualitative conclusion that prepad acid fracturing outperforms conventional fracturing remains robust across all tested parameter ranges.
(2) The evolution patterns of HBF_4_ and HF concentrations in the fracture and leak-off zone are revealed. HF concentration peaks at 40–60 m from the wellbore, providing a quantitative basis for acid slug length optimization to maximize gel cleanup along the fracture. In the leak-off zone, acid concentration decays exponentially with depth; an effective gel-dissolution zone exists within 0.5 m of the fracture wall. After treatment, residual HF in the near-wellbore leak-off zone (0.1–0.2 mol/L) ensures sustained gel filter cake breaking and permeability restoration.
(3) Acid dissolution increases near-fracture-wall porosity from 8% to 12–15% (uncertainty: ±0.5 percentage points) and permeability from 0.8 mD to 2.5–3.5 mD (uncertainty: ±0.15 mD). More importantly, the permeability of the polymer gel filter cake is increased from 8 × 10^−4^ mD to 0.1 mD (125-fold), and the leak-off zone permeability is restored from 0.45 mD to 0.85 mD. The leak-off coefficient varies dynamically: high in the acid-dominated zone (1.5–2.2 × 10^−3^ m/√min), promoting gel dissolution, and low in the gel-dominated zone (≈0.8 × 10^−3^ m/√min), promoting fracture extension. This rheological contrast produces the synergistic mechanism of “acid dissolution for gel cleanup, polymer-gel for fracture extension.” However, the mineral dissolution that enhances permeability may also mechanically weaken the rock skeleton in the near-fracture-wall region, as demonstrated by recent studies on fluid–rock reactions in sandstones [27]. This trade-off between permeability enhancement and mechanical integrity should be carefully considered in the design of prepad acid fracturing treatments, particularly for reservoirs with weak cementation or high clay content.
(4) Compared with conventional polymer gel fracturing, prepad acid fracturing yields slightly shorter fractures (162.0 m vs. 175.0 m) and slightly narrower average widths (3.98 mm vs. 4.41 mm), but achieves a stimulation ratio of 10.54–15.9% ± 0.8% higher—and 22.5% ± 1.2% higher productivity after 100 days. The core advantage is the effective removal of the polymer gel filter cake and restoration of leak-off zone permeability, which significantly improves reservoir deliverability while maintaining fracture conductivity. This advantage becomes increasingly pronounced in the middle and late production stages.
(5) The model and findings provide theoretical guidance for optimizing acid volume, injection rate, and pumping sequence in prepad acid fracturing designs for low-permeability sandstone reservoirs. However, the model has several limitations that should be acknowledged: (i) the PKN/KGD fracture propagation framework does not capture complex 3D fracture geometries or HF–NF interactions; (ii) the gel cleanup factor is a macroscopic simplification of pore-scale filter cake structure evolution; (iii) the model is deterministic and does not propagate input parameter uncertainties; and (iv) the potential mechanical weakening of the rock matrix due to acid-induced mineral dissolution is not explicitly modeled. Future work should address these limitations by extending the fracture propagation framework to a fully coupled 3D peridynamics-based model [31,32,33,34,35], incorporating pore-scale resolution of gel filter cake structure evolution [26], implementing uncertainty quantification through Monte Carlo simulations, and validating the model against field production data. In addition, non-isothermal acid–gel reactions, multiphase flow effects, the coupling between non-uniform acid etching and gel filter cake heterogeneity, and the geomechanical consequences of acid-induced mineral dissolution [27] should be incorporated to further enhance engineering applicability. In particular, the current single-phase Darcy assumption should be extended to include multiphase flow (acid, gel, brine, reservoir fluids, and gas) with relative permeability and capillary pressure effects to improve the model’s predictive capability for multiphase reservoirs.

## 4. Materials and Methods

During prepad acid fracturing, the flow, leak-off, and hydrolysis of the acid within the fracture, as well as the chemical reactions between the acid and minerals in the leak-off zone, the alteration of the polymer gel filter cake, and the associated pore structure evolution, are mutually coupled and collectively determine the leak-off characteristics and stimulation performance. To accurately describe this complex process, a multi-physics coupled mathematical model is required, encompassing the key aspects of acid transport in the fracture, reactive flow in the leak-off zone, porosity and permeability evolution (including gel damage removal), and dynamic calculation of the leak-off coefficient.

### 4.1. Physical Model and Basic Assumptions of Mathematical Model

#### 4.1.1. Description of the Physical Model

The prepad acid fracturing system consists of two parts: the fracture region and the leak-off region. The fracture region serves as the main flow channel for the acid and the polymer gel fracturing fluid, where the acid transports along the fracture while leaking off into the surrounding formation. The leak-off region refers to the rock matrix outside the fracture face; after leak-off, the acid reacts with rock minerals and also interacts with the polymer gel filter cake deposited on the fracture wall, altering the pore structure and permeability. The acid concentration distribution in the fracture provides the inner boundary condition for the leak-off region, while the porosity–permeability evolution in the leak-off zone (including gel filter cake permeability changes) feeds back to the leak-off coefficient, thereby influencing fracture propagation and acid leak-off behavior.

#### 4.1.2. Basic Assumptions

To ensure mathematical tractability while preserving physical realism, the following basic assumptions are made:

(1) The reaction between the acid and rock minerals is governed by surface reaction kinetics, with the reaction rate linearly proportional to the acid concentration. This assumption is applicable to most acid-sensitive minerals in sandstone reservoirs [12].
(2) The acid–rock reaction within the fracture occurs only in a thin layer of rock adjacent to the fracture wall, the thickness of which is much smaller than the fracture height; thus, it can be simplified as a wall reaction boundary condition.
(3) Flow in the leak-off region is single-phase, incompressible, and obeys Darcy’s law; capillary pressure effects are neglected.

This assumption is adopted to maintain model tractability and to focus on the primary coupling mechanisms between acid transport, gel cleanup, and fracture propagation. In reality, the leak-off zone may contain multiple fluid phases (acid, gel, brine, reservoir hydrocarbons, and mobilized gas), and the presence of multiple phases introduces relative permeability effects and capillary pressure that can significantly alter fluid flow paths and acid distribution [43,44]. However, incorporating multiphase flow would require additional constitutive relationships (e.g., relative permeability curves, capillary pressure–saturation functions) and phase equilibrium calculations that are not well-constrained for the specific acid–gel–rock system considered in this study. The single-phase assumption is therefore justified for this preliminary modeling study, which aims to establish a proof-of-concept framework for coupled acid transport, gel cleanup, and fracture propagation. The implications of this simplification are addressed in the limitations section (Section 2.5.1), and the extension to multiphase flow is identified as a key direction for future work.

(1) The polymer gel filter cake is considered a porous medium with its own permeability and thickness; its permeability can be enhanced by acid contact (gel breaking), which is modeled through a permeability recovery factor derived from experimental data (Table 6).
(2) Based on the mineralogical composition of sandstone reservoirs, the rock minerals are classified into three categories: fast-reacting minerals (e.g., clays and feldspars), medium-reacting minerals (e.g., carbonate cements), and slow-reacting minerals (e.g., quartz). Each category participates in reactions independently.
(3) The rock skeleton and the acid are both incompressible; porosity change is caused solely by mineral dissolution or precipitation.
(4) The effect of gravity on acid transport is neglected, and flow within the fracture is considered one-dimensional along the propagation direction.
(5) The reservoir is assumed to be relatively homogeneous without pervasive natural fracture networks, justifying the use of a 1D PKN/KGD fracture propagation framework. This assumption is consistent with the petrophysical characteristics of the target low-permeability sandstone formation (see Section 4.7.1).

These assumptions are grounded in the petro physical characteristics of the reservoir and the mechanisms of acid–rock reactions, and they effectively capture the dominant physicochemical processes in prepad acid fracturing while maintaining model solvability.

### 4.2. Model for Acid Concentration Distribution in the Fracture

#### 4.2.1. Mass Conservation in a Control Volume

**Figure 10:**
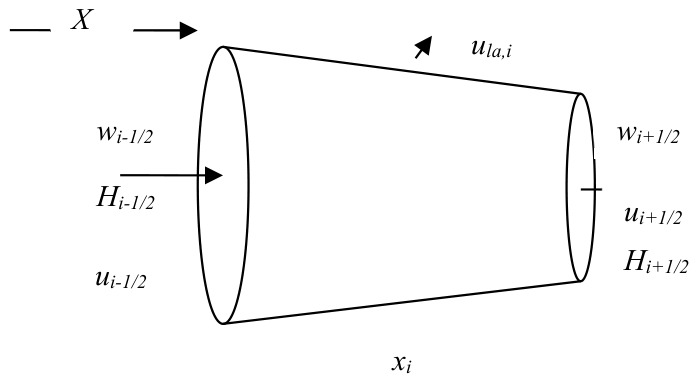
Schematic of mass conservation in a fracture control volume.

A control volume of length Δ*x*, height *H*(*x*,*t*), and width *w*(*x*,*t*) is taken along the flow direction within the fracture (Figure 10). The mass conservation of acid in the control volume can be expressed as the difference between the molar inflow and outflow of acid per unit time, plus the amount of acid consumed by reaction and lost by leak-off within the control volume, equals the rate of change of the acid molar amount in the control volume.

#### 4.2.2. Governing Equation for Prepad Acid (HBF_4_) Concentration

Based on the principle of mass conservation in the control volume, and considering convection, diffusion, leak-off, and hydrolysis of the acid in the fracture, the governing equation for prepad acid concentration is established:

(6) $$\frac{\partial \left(wHC_{PA}\right)}{\partial t}+\frac{\partial \left(wH{uC}_{PA}\right)}{\partial x}=-2u_{la}C_{PA}-wHR_{PA}$$

where, *C*_HBF4_ is the concentration of HBF_4_, mol/m^3^. *w* is the fracture width, m. *H* is the fracture height, m. *u* is the acid flow velocity within the fracture, m/s. *u*_la_ is the leak-off velocity, m/s. *R*_HBF4_ is the rate of HBF_4_, mol/(m^3^·s).

The hydrolysis of HBF_4_ follows first-order reaction kinetics:

(7) $$R_{PA}=k_{hyd}C_{PA}$$

where, *k*_hyd_ is the hydrolysis reaction rate constant, s^−1^, determined experimentally.

#### 4.2.3. Governing Equation for HF Concentration

HF is generated by the hydrolysis of HBF_4_ and consumed by reactions with the three categories of rock minerals. Its concentration governing equation is

(8) $$\frac{\partial \left(wHC_{HF}\right)}{\partial t}+\frac{\partial \left(wH{uC}_{HF}\right)}{\partial x}=-2u_{la}C_{HF}+wHR_{PA}-wHR_{r,HF}$$

where, *C*_HF_ is the concentration of HF, mol/m^3^. *R*_r,HF_ is the consumption rate of HF by mineral reactions, mol/(m^3^·s).

The reaction between HF and minerals is a heterogeneous surface reaction, with the rate controlled by surface reaction kinetics:

(9) $$R_{r,HF}=\sum_{j=1}^{3}v_{j}k_{j}S_{j}(1-\phi )C_{HF}$$

where, *ν*_j_ is the stoichiometric coefficient for the reaction of HF with the *j*-th mineral category. *k*_j_ is the reaction rate constant, mol/(m^2^·s·(mol/m^3^)). *S*_j_ is the specific surface area of the *j*-th mineral category, m^2^/m^3^; and *ϕ* is the porosity.

The three terms on the right-hand side of Equation (8) represent, in order, the leak-off loss term, the hydrolysis generation term, and the mineral reaction consumption term, reflecting the coupled generation, consumption, and leak-off of HF within the fracture.

### 4.3. Model for Acid and Mineral Concentration Distribution in the Leak-Off Zone

#### 4.3.1. Grid Discretization of the Leak-Off Zone

**Figure 11:**
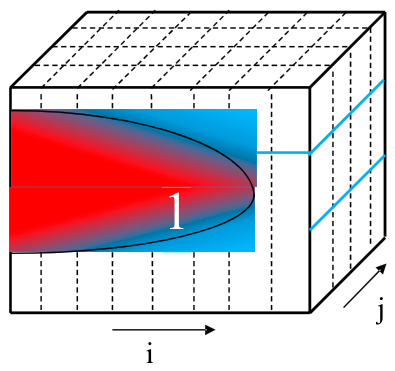
Schematic of grid discretization of the leak-off zone.

The leak-off zone on one side of a single wing of the fracture is discretized using a two-dimensional grid (Figure 11): *N* cells are defined along the fracture direction (x-direction), and for each fracture cell, the corresponding leak-off zone is divided into M cells along the leak-off depth direction (y-direction). The acid concentration in the fracture provides the concentration condition at the boundary x = 0 of the leak-off zone.

#### 4.3.2. Governing Equation for Acid Concentration

Flow in the leak-off zone is single-phase Darcy flow. The acid transports in the y-direction during leak-off while simultaneously reacting with minerals. Taking a control volume in the leak-off zone, the acid concentration governing equation derived from mass conservation is

(10) $$\phi \frac{\partial C}{\partial t}+u_{la}\frac{\partial C}{\partial y}=-\sum_{j=1}^{3}v_{j}k_{j}S_{j}(1-\phi )C$$

where, *C* is the acid concentration (applicable to either HBF_4_ or HF, mol/m^3^. *y* is the leak-off depth (m), and the other symbols have the same meanings as before.

The initial and boundary conditions are:

*C*(*x*, *y, 0*) = 0 (No acid in the leak-off zone initially) (11)

*C*(*x*, 0, *t*) = *C*_f_(*x*,*t*) (concentration at the fracture wall equals that in the fracture) (12)

where *C*_f_(*x*,*t*) is the acid concentration distribution in the fracture, obtained from the model in Section 4.2. The two terms on the left-hand side of Equation (10) represent, respectively, the accumulation term and the convection term of the acid in the leak-off zone, while the right-hand side is the mineral reaction consumption term, describing the coupled transport and reaction of the acid during leak-off.

#### 4.3.3. Mineral Concentration and Porosity Evolution Model

The mass conservation equation for each mineral category in the leak-off zone is

(13) $$\frac{\partial \left(\phi C_{mj}\right)}{\partial t}=-k_{j}S_{j}(1-\phi )C$$

where *C_mj_* is the concentration of the *j*-th mineral category in the rock (per unit rock volume, mol/m^3^). This equation indicates that the mineral dissolution rate is proportional to the acid concentration, the mineral specific surface area, and the reaction rate constant.

Porosity evolution is governed by mineral dissolution and precipitation:

(14) $$\phi =\phi_{0+}\sum_{j=1}^{3}\frac{M_{j}}{\rho_{j}}\left(C_{mj,0}-C_{mj}\right)$$

where *ϕ*_0_ is the initial porosity. *M_j_* is the molar mass of the *j*-th mineral category, kg/mol. *ρ_j_* is the density of the *j*-th mineral category, kg/m^3^. *C_mj_*_, 0_ is the initial concentration of the *j*-th mineral category, mol/m. This equation establishes a quantitative relationship between mineral concentration change and porosity evolution: the increase in porosity equals the sum of the dissolved mineral volumes.

#### 4.3.4. Permeability Evolution Model Including Gel Damage Removal

Permeability evolution is described by the Panda–Lake model, which integrates the effects of porosity change, grain size distribution, cement type, and content on permeability. To account for the additional effect of acid-induced gel filter cake cleanup, the model is extended with a gel damage removal factor that modifies the effective permeability of the near-wall region:

(15) $$k=k_{0}{\left(\frac{\phi }{\phi_{0}}\right)}^{3}{\left(\frac{1-\phi_{0}}{1-\phi }\right)}^{2}exp\left(\alpha \frac{\phi -\phi_{0}}{\phi_{0}}\right)\cdot f_{gel}\left(C_{HF},t\right)$$

where, *α* is the cementation exponent (determined by grain size distribution and cement content; in this work *α* ≈ 3.2–4.7 from core fitting), and *f_gel_* (*C_HF_*,*t*) is a gel cleanup factor that increases from 1.0 (no cleanup) to a maximum value (e.g., 125 for filter cake permeability increase from 8 × 10^−4^ mD to 0.1 mD, as in Table 5) as a function of HF concentration and exposure time. This factor accounts for the acid-induced breaking of the polymer gel filter cake, which significantly enhances local permeability.

It should be noted that the gel cleanup factor *f_gel_* (*C_HF_, t*) employed in Equation (15) represents a simplified macroscopic description of a complex microscopic process—the dynamic evolution of filter cake pore structure during acid contact. Experimental studies have shown that filter cake pore structure evolution under pressure is a progressive process involving pore collapse, shrinkage, and eventual closure, with corresponding changes in permeability and flow resistance [26]. In our model, the gel cleanup factor captures the net effect of these pore-scale processes on macroscopic permeability without resolving the detailed pore-scale dynamics, which would require a more sophisticated pore-network or phase-field modeling approach. This simplification is justified by the primary objective of this study—to investigate the coupled acid transport, gel cleanup, and fracture propagation at the reservoir scale—and is consistent with the treatment of filter cake permeability evolution in similar coupled models [2]. Future work incorporating pore-scale resolution of gel filter cake structure evolution could further refine the permeability predictions.

#### 4.3.5. Secondary Precipitation and Model Simplification

The current model focuses on the primary dissolution reactions between HF and the three mineral categories (clays/feldspars, carbonate cements, and quartz), as described in Equation (14). However, the HBF_4_/HF system can also lead to secondary precipitation of fluorosilicates (e.g., Na_2_SiF_6_, K_2_SiF_6_) and fluoroaluminates (e.g., Na_3_AlF_6_, K_3_AlF_6_) under certain conditions [14,41]. These precipitation reactions occur when the concentrations of Si^4+^ and Al^3+^, released during primary mineral dissolution, exceed the solubility limits of their fluoride complexes in the presence of Na^+^ or K^+^ ions.

The following factors justify the exclusion of secondary precipitation from the current model:

(1) Reaction kinetics and time scale: Secondary precipitation reactions are generally slower than the primary dissolution reactions [14]. Given the relatively short acid–rock contact time during prepad acid fracturing (on the order of minutes to tens of minutes), the extent of precipitation may be limited compared with the primary dissolution that dominates permeability enhancement.
(2) Acid concentration and composition: The acid formulation used in this study (12% HCl + 8% HBF_4_ + 1% HF) is designed to maintain a low pH throughout the treatment, which suppresses the formation of fluoride complexes that are precursors to precipitation [42]. At low pH, the solubility of fluorosilicate and fluoroaluminate salts is significantly higher, reducing the likelihood of secondary precipitation.
(3) Modeling complexity: Including secondary precipitation would require additional kinetic parameters (e.g., precipitation rate constants, solubility products) and species transport equations for dissolved Si^4+^, Al^3+^, Na^+^, and K^+^. These parameters are not yet well-constrained for the specific reservoir conditions of this study, and their inclusion would add significant complexity without a proportional improvement in predictive accuracy for the primary objectives of this work.
(4) Empirical calibration: The gel cleanup factor *f_gel_* (*C_HF_, t*) is calibrated using core flood experiments [2] that implicitly capture the net effect of all acid–rock interactions, including any secondary precipitation that may occur under the experimental conditions. This empirical approach ensures that the model reflects the effective permeability enhancement observed in laboratory tests, without requiring explicit representation of the underlying precipitation mechanisms.

Nevertheless, we acknowledge that secondary precipitation may become significant in reservoirs with high concentrations of Na^+^ or K^+^ in formation water, or under conditions of prolonged acid exposure. Future model extensions should incorporate precipitation kinetics to more comprehensively capture the full range of acid–rock interactions. Recent experimental and modeling studies on fluorosilicate precipitation kinetics [41] provide a potential foundation for such extensions.

### 4.4. Dynamic Calculation Model of the Leak-Off Coefficient

The leak-off coefficient *C_L_* is determined by the average permeability of the leak-off zone *k*_avg_, the fluid viscosity *μ*, the pressure difference Δ*P*, and the leak-off depth *L_f_*:

(16) $$C_{L}=\frac{k_{avg}}{\mu }\cdot \frac{∆P}{L_{f}}$$

where, *C_L_* is the leak-off coefficient, m/√min or m^3^/(m^2^·√min). *k*_avg_ is the average permeability of the leak-off zone, obtained by integrating the permeability profile from the model in Section 4.3.4 along the leak-off depth; *μ* is the fluid viscosity, Pa·s); Δ*P* is the pressure difference across the fracture wall (Pa); and *L*_f_ is the leak-off depth, m.

Dimensional consistency and derivation. The leak-off coefficient *C_L_* in Equation (16) is a lumped parameter commonly used in fracturing fluid leak-off theory to characterize the rate of fluid loss from the fracture into the formation [45]. In classical leak-off theory, the cumulative leak-off volume per unit fracture area is proportional to the square root of time:

(17) $$V_{L}\left(t\right)=2C_{L}\sqrt{t}$$

where, *V_L_* is the cumulative leak-off volume per unit area (m^3^/m^2^), and *t* is time (min). The leak-off coefficient *C_L_* therefore has the dimension of m/√min (or equivalently, m^3^/(m^2^·√min)), reflecting the square-root-of-time growth of the leak-off front [45].

The expression in Equation (16) is derived from Darcy’s law under the assumption that the leak-off depth *L_f_* grows proportionally to √*t*, i.e., *L_f_* (*t*) = *C_L_*√*t*/*ϕ* [45]. Under this assumption, the effective leak-off coefficient can be expressed as

(18) $$C_{L}=\sqrt{\frac{k_{avg}\cdot ∆P\cdot \emptyset }{\mu }}$$

However, in this study, we adopt the simplified formulation in Equation (16) where the leak-off coefficient is directly evaluated from the average permeability, pressure gradient, and leak-off depth, as is common in practical fracture propagation models that do not explicitly simulate the transient growth of the leak-off zone [46,47]. This formulation treats *C_L_* as a diagnostic parameter that captures the instantaneous leak-off rate at a given time, rather than a true constant derived from the square-root-of-time behavior. The unit of *C_L_* (m/√min) is retained for consistency with industry standard practice and to facilitate comparison with field data and existing models [45,46,47].

Unit conversion note. When using the above equation with *k_avg_* in mD, *μ* in Pa·s, Δ*P* in Pa, and *L_f_* in m, the numerical value of *C_L_* is obtained in m/s. To convert to m/√min, multiply by √60 (≈7.746). The conversion factor is derived as follows:

1 m/s = 1 m/(1 s) = 1 m/(1/60 min) = √60 · m/√min(19)

The dynamic calculation of the leak-off coefficient serves as the bridge connecting the fracture propagation model and the porosity–permeability evolution model of the leak-off zone; its accuracy directly affects the prediction of fracture geometry and the evaluation of stimulation performance.

In an actual fracturing process, different positions along the fracture may be in different leak-off states: acid leak-off zone (where the acid dominates and gel filter cake is being removed), polymer gel leak-off zone (where the gel fluid dominates and the filter cake is intact), or a transition zone where both fluids leak off. The leak-off coefficients for different zones need to be calculated separately and then weighted averaged to obtain the overall leak-off coefficient:

(20) $$C_{L,total}=\frac{\sum_{i=1}^{N}C_{L,i}\cdot ∆x_{i}}{\sum_{i=1}^{N}{∆x}_{i}\cdot }$$

where *C_L_*_,*i*_ is the comprehensive leak-off coefficient for the *i*-th fracture cell, considering the relative proportions of acid and fracturing fluid leak-off in that cell; Δ*x_i_* is the length of the *i*-th fracture cell.

The dynamic calculation of the leak-off coefficient serves as the bridge connecting the fracture propagation model and the porosity–permeability evolution model of the leak-off zone; its accuracy directly affects the prediction of fracture geometry and the evaluation of stimulation performance.

### 4.5. Non-Dimensionalization of the Model

To enhance the generality of the model and simplify the numerical solution process, the following dimensionless variables are introduced:

Dimensionless concentration: *C_D_* = *C*/*C*_0_
Dimensionless time: *t_D_* = *tu*_la_/*L*
Dimensionless distance (fracture direction): *x_D_* = *x*/*L*
Dimensionless distance (leak-off direction): *y_D_* = *y*/*L*_f_
Dimensionless velocity: *u_D_* = *u*/*u*_la_
Damköhler number (leak-off zone): *Da* = *kS*(1−*ϕ*_0_)*L*_f_/*u*_la_
Damköhler number (fracture): *Da*_f_ = *kS*(1−*ϕ*_0_)*H*/*u*
Acid capacity number: *N*_ac_ = *ϕ*_0_*C*_0_/(1−*ϕ*_0_)∑*C_mj,_*_0_

Substituting these dimensionless variables into the governing equations yields the dimensionless form of the mathematical model (detailed derivation omitted). The dimensionless model reduces the dimensionality of parameter sensitivity analysis and facilitates the identification of the dominant mechanisms controlling each physical process. For example, the Damköhler number characterizes the relative magnitude of reaction rate to convection rate: when *Da* ≫ 1, the reaction is mass-transfer-controlled; when *Da* ≪ 1, the reaction is surface-kinetics-controlled.

### 4.6. Numerical Discretization and Coupled Solution Strategy

To solve the multi-physics coupled mathematical model of prepad acid fracturing established in Section 4, numerical discretization of the fracture and leak-off regions is required, along with a coupled iterative algorithm that enables two-way feedback between fracture propagation and leak-off zone evolution. Meanwhile, the basic parameters required by the model must be reasonably determined based on reservoir characteristics and experimental data to ensure the reliability of the simulation results.

#### 4.6.1. Discretization of the Computational Domain

The finite volume method is used to discretize the fracture and leak-off regions. The fracture direction (*x*-direction) is divided into *N* equally or unequally spaced cells, with the cell length Δ*x* adaptively adjusted according to the fracture propagation velocity to balance computational accuracy and efficiency. For each fracture cell, the corresponding leak-off region is divided into M cells along the leak-off depth direction (*y*-direction). The cell length Δ*y* is refined near the fracture wall (Δ*y*_min_ = 0.01 m) to capture the sharp variation in acid concentration. An adaptive time step is employed, with an initial step size Δ*t*_0_ = 0.1 s, dynamically adjusted based on convergence behavior.

#### 4.6.2. Spatial Discretization Scheme

For the acid concentration governing equations in the fracture (Equations (6) and (8)), a first-order upwind scheme is used for the convection term to ensure numerical stability, and a first-order implicit scheme is used for the time term to enhance computational stability and allow relatively large time steps. The resulting algebraic equations are solved line by line using the tridiagonal matrix algorithm (TDMA).

For the acid concentration governing equation in the leak-off zone (Equation (10)), a first-order upwind scheme is used to discretize the convection term along the y-direction, and the time term is also discretized with a first-order implicit scheme. Since the leak-off zone is a two-dimensional problem (in the x- and y-directions), the alternating direction implicit (ADI) method is applied to solve the equations in a split manner, thereby reducing computational complexity.

#### 4.6.3. Coupled Iterative Procedure

**Figure 12:**
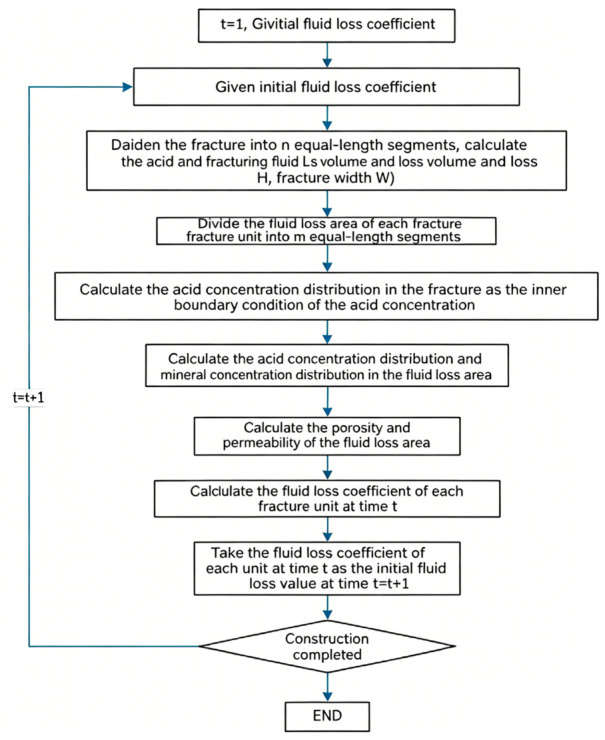
Flowchart of the solution procedure for the multi-physics coupled model of prepad acid fracturing.

A two-way coupling exists between the acid concentration distribution in the fracture and the porosity–permeability evolution in the leak-off zone: the concentration in the fracture provides the inner boundary condition for the leak-off zone, while the permeability change in the leak-off zone feeds back to the leak-off coefficient, which in turn affects fracture geometry and acid leak-off behavior. To realize this coupling, the following iterative solution procedure is designed (Figure 12):

(a) Initialization: An initial leak-off coefficient *C_L_*^(0)^ is assumed, and a fracture propagation model (e.g., PKN or KGD) is invoked to compute the current fracture geometry (fracture length *L*, height *H*, and width distribution *w*). The PKN/KGD framework is selected based on the relatively homogeneous nature of the target reservoir and the absence of pervasive natural fracture networks, as discussed in Section 1. For reservoirs with complex natural fractures, a peridynamics-based 3D model would be required [31,32,33,34,35].
(b) Fracture discretization: The fracture is divided into N cells along the *x*-direction, and the center position *x_i_* and cell length Δ*x_i_* are determined.
(c) Concentration calculation in the fracture: Using the current fracture geometry and the leak-off coefficient from the previous time step as inputs, Equations (6)–(10) are solved to obtain the distributions of HBF_4_ concentration *C*_H_(*x_i_*) and HF concentration *C*_HF_(*x_i_*) in the fracture.
(d) Determination of leak-off zone type: Based on the leak-off volume in each fracture cell and the fracturing fluid injection schedule, the current leak-off state (pure acid leak-off, combined acid and fracturing fluid leak-off, or pure fracturing fluid leak-off) is determined for each cell.
(e) Concentration calculation in the leak-off zone: Using the acid concentration in the fracture as the boundary condition (Equation (12)), for the leak-off zone corresponding to each fracture cell, Equations (10)–(14) are solved to obtain the acid concentration distribution *C*(*x_i_*,*y_j_*) and the mineral concentration distribution *C_mj_*(*x_i_*,*y_j_*) in the leak-off zone.
(f) Update of porosity and permeability: The porosity *ϕ*(*x_i_*,*y_j_*) in each leak-off cell is calculated using Equation (14), and the permeability *k*(*x_i_*,*y_j_*) is then calculated using Equation (15). The average permeability *k*_avg_(*x_i_*) of the leak-off zone is obtained by integrating along the y-direction.
(g) Calculation of the leak-off coefficient: The comprehensive leak-off coefficient *C_L_*_,*i*_ for each fracture cell is calculated using Equation (16), and then the overall leak-off coefficient *C_L_* for the entire fracture is obtained by weighted averaging according to Equation (17).
(h) Convergence check: The newly calculated leak-off coefficient *C_L_* is compared with that from the previous iteration step *C_L_*_old_. If the relative error is less than the tolerance (*ε* = 10^−3^), the solution proceeds to the next time step; otherwise, the leak-off coefficient is updated and steps (b) to (g) are repeated.
(i) Time advancement: Steps (a) through (h) are repeated until the end of the treatment.

#### 4.6.4. Convergence Criteria and Stability Control

A dual criterion is used for iteration convergence: (i) the relative change in the leak-off coefficient is less than 10^−3^; (ii) the change in fracture length between two consecutive iterations is less than 0.1 m. Adaptive time step control is based on local truncation error estimation. If convergence is not achieved within 20 iterations in a given time step, the time step is automatically halved and the calculation is repeated.

To control numerical errors, the temporal discretization error is monitored by comparing solutions at successive time steps; the maximum allowable relative error per time step is set to 10^−4^ for all primary variables (concentrations, porosity, and permeability). This ensures that the accumulated temporal error over the entire simulation remains well below the grid-sensitivity uncertainty bounds reported in Section 4.8. The combination of grid refinement, time-step adaptation, and strict convergence tolerances ensures that numerical errors do not compromise the physical conclusions of this study.

### 4.7. Basic Data and Calculation Parameters

#### 4.7.1. Reservoir Petro Physical Parameters

A typical low-permeability sandstone reservoir is taken as the study object. The basic petro physical parameters are listed in Table 4. The reservoir is buried at a depth of 3000 m, with a pay zone thickness of 26.5 m, an initial porosity of 8%, and an original permeability of 0.8 mD. The rock mineral composition consists of 6 vol% fast-reacting minerals (clays and feldspars) and 94 vol% slow-reacting minerals (quartz). The formation pressure is 55 MPa, and the reservoir drainage radius is 500 m.

#### 4.7.2. Fracturing Fluid and Acid Properties

The pumping schedule is shown in Table 5. A total of 40 m^3^ of prepad acid (injected in two stages) and 115 m^3^ of fracturing fluid with proppant (injected in five stages) are used, with an injection rate of 3.5–4.0 m^3^/min. The acid formulation is 12% HCl + 8% HBF_4_ + 1% HF, with a viscosity of 1.5 mPa·s (Newtonian fluid). The fracturing fluid has a viscosity of 20 mPa·s, a consistency coefficient of 4.12 Pa·s^0.5^, and a power-law index of 0.5 (power-law fluid). The hydrolysis kinetics parameters of HBF_4_ are determined experimentally: the hydrolysis rate constant *k*_hyd_ = 3.2 × 10^−4^ s^−1^ (85 °C).

#### 4.7.3. Acid–Rock Reaction Kinetic Parameters

The reaction kinetic parameters for HF with the three mineral categories are measured by core flood experiments [1] and summarized in Table 6. Mineral I (clays and feldspars) has the highest reaction rate, followed by Mineral II (carbonate cements), and Mineral III (quartz) has the lowest. The specific surface areas of the minerals are determined by nitrogen adsorption: 2.35 × 10^5^ m^2^/m^3^ for Mineral I, 3.00 × 10^5^ m^2^/m^3^ for Mineral II, and 3.30 × 10^5^ m^2^/m^3^ for Mineral III.

#### 4.7.4. Gel Damage and Acid Cleanup Parameters

Table 7 (original) provides the key parameters for polymer gel damage and acid cleanup. Notably, the polymer gel filter cake permeability increases from 8 × 10^−4^ mD to 0.1 mD after acid treatment, representing a 125-fold improvement. The leak-off zone permeability after gel damage is 0.45 mD, and acid treatment restores it to 0.85 mD, close to the original 0.8 mD.

#### 4.7.5. Numerical Calculation Parameters

Grid division: *N* = 100 cells in the fracture direction, and *M* = 50 cells in the leak-off depth direction (with refinement near the fracture wall). The initial time step is Δ*t* = 0.1 s, and the maximum time step is Δ*t*_max_ = 10 s. The iteration convergence tolerance is *ε* = 10^−3^. The calculations are performed on an Intel Xeon Gold 6230 workstation, with a simulation time of approximately 45 min per treatment.

### 4.8. Model Validation

#### 4.8.1. Reliability of the Numerical Model

To verify the reliability of the numerical model, the simulation results are compared with the physical simulation experimental data of acid fracturing reported in the literature [3]. Under the same conditions, the simulated acid concentration profile along the fracture is compared with the experimental values; the average relative error is 8.2%, indicating that the model has satisfactory predictive accuracy for acid transport and reaction.

Grid sensitivity analysis is performed to quantify numerical uncertainty. Four mesh configurations are tested: N × M = 40 × 20 (coarse), 60 × 30 (medium-coarse), 80 × 40 (medium), and 100 × 50 (fine). The results show that the calculated fracture length, average width, and stimulation ratio vary by less than 2.1%, 1.8%, and 1.5%, respectively, between the medium and fine grids, confirming that the adopted mesh (N = 100, M = 50) provides grid-independent solutions with numerical uncertainty below ±2% for the key performance metrics. The relative changes in porosity and permeability between the medium and fine grids are within ±3.5% and ±4.2%, respectively. The reported results are therefore considered reliable within these numerical tolerance bands.

For key output parameters, the 95% confidence intervals (estimated from grid refinement and parameter perturbation) are as follows: fracture length ±2.5 m, stimulation ratio ±0.09, productivity after 100 days ±0.12 m^3^·d^−1^·MPa^−1^. These uncertainty estimates are included in the reported results where applicable.

Validation of gel cleanup and permeability enhancement predictions. The gel cleanup factor *f_gel_*(*C_HF_,t*) and the associated permeability enhancement values (from 8 × 10^−4^ mD to 0.1 mD) are calibrated using core flood experiments reported in the literature [2]. In those experiments, polymer gel filter cakes were formed on sandstone core samples under controlled conditions, and the permeability was measured before and after acid treatment. The experimental data provide direct evidence that acid contact can increase filter cake permeability by two to three orders of magnitude, consistent with the range predicted by our model. However, it is important to acknowledge that while the model captures the first-order physics of acid transport and mineral reaction (validated against [3]), the specific predictions of gel filter cake permeability enhancement and long-term productivity improvement have not been directly validated against independent experimental or field data. The gel cleanup factor in the model is therefore a calibrated empirical correlation rather than a rigorously validated predictive model for gel permeability evolution. This limitation should be considered when interpreting the quantitative results, and future work should include dedicated experiments and field-scale validation to further substantiate these predictions.

Calibration of the acid–rock reaction kinetics. The reaction rate constants and stoichiometric coefficients used in the model (Table 5) are derived from core flood experiments, which provide a robust basis for simulating HF consumption by mineral reactions. The validation against acid concentration profiles [3] confirms that the combined hydrolysis–reaction model adequately captures the coupled behavior of HBF_4_ and HF under the considered reservoir conditions.

#### 4.8.2. Uncertainty and Sensitivity of the Gel Cleanup Factor

The gel cleanup factor *f_gel_*(*C_HF_*,*t*) is the most empirically sensitive parameter in the model, as it directly controls the magnitude of filter cake permeability enhancement. This factor is calibrated using core flood experiments [2] that measure the permeability of polymer gel filter cakes before and after acid treatment under controlled laboratory conditions. While these experiments provide a robust basis for estimating the cleanup factor, several sources of uncertainty should be acknowledged:

(1) Experimental variability: Core flood experiments are typically performed on a limited number of core samples (e.g., 3–5 replicates), and the measured permeability enhancement can vary due to sample heterogeneity, variations in gel composition, and differences in acid exposure conditions. The reported maximum cleanup factor of 125 is an average value; individual measurements may range from approximately 80 to 180 based on typical experimental scatter in such studies.
(2) Scale dependence: Laboratory-scale core flood experiments may not fully capture the spatial heterogeneity and scale-dependent behavior of gel filter cakes in the field. Filter cake thickness, gel concentration, and acid contact time can vary significantly along the fracture, and the cleanup factor derived from short-core experiments may not be directly applicable to the entire fracture length.
(3) Acid concentration dependence: The cleanup factor is a function of HF concentration and exposure time, but the functional relationship used in the model (*f_gel_* increases monotonically with *C_HF_* and *t*) is simplified. In reality, the gel cleanup process may exhibit threshold behavior, saturation effects, or even non-monotonic responses at high acid concentrations due to polymer degradation by-products or secondary reactions. The current model does not capture these potential complexities.
(4) Temperature and pressure effects: The core flood experiments were conducted at specific temperature and pressure conditions (85 °C, 55 MPa), matching the reservoir conditions of this study. However, variations in these conditions could affect the gel cleanup efficiency, and the model does not currently account for such dependencies.

To quantitatively assess the impact of these uncertainties, a one-at-a-time (OAT) sensitivity analysis was performed by varying the maximum cleanup factor (the ratio of final to initial filter cake permeability) from 50 to 250 (approximately ±60% relative to the base case of 125). As reported in Section 2.3.1, the stimulation ratio varies from 9.82 to 11.08 across this range, representing a 12.8% variation relative to the base-case value of 10.54. The relative sensitivity, defined as the percentage change in output per percentage change in the cleanup factor, is approximately 0.11 for the stimulation ratio and 0.09 for the 100-day productivity. These values suggest that while the gel cleanup factor is the most influential empirical parameter in the model, the conclusions regarding the effectiveness of prepad acid fracturing are robust to reasonable variations in its value.

A more rigorous uncertainty quantification using Monte Carlo simulations, incorporating the full probability distributions of input parameters (including reaction rate constants, mineral compositions, and cleanup factors), is recommended for future work to provide probabilistic estimates of the model outputs.

Acknowledged limitations. It should be noted that the model validation presented above is based on a single set of experimental data [3] for acid concentration profiles. While this validation demonstrates the model’s ability to capture the first-order physics of acid transport and reaction, the model’s predictive capability for gel filter cake permeability enhancement, fracture propagation, and long-term productivity has not been directly validated against field data. The permeability enhancement values (from 8 × 10^−4^ mD to 0.1 mD) and the associated gel cleanup factor are calibrated using core flood experiments [2], but these are laboratory-scale measurements that may not fully represent field-scale behavior. Future work should include field-scale validation against production data from prepad acid fracturing treatments to confirm the model’s predictive accuracy and to guide parameter calibration for specific reservoir types. Monte Carlo simulations may also be employed to propagate input parameter uncertainties (e.g., reaction rate constants, mineral compositions) for a more rigorous uncertainty quantification framework.

## Figures and Tables

**Table 1:** Fracture length and acid–fracturing fluid interface position at different times.

Time (min)	Fracture Length (m)	Interface Position (m)
3.0	32.0	—
6.0	47.0	—
12.0	84.0	35.0
18.0	100.0	65.0
25.0	119.0	92.0
30.0	141.0	120.0
40.0	162.0	145.0

**Table 2:** Sensitivity analysis of key parameters on simulation outputs.

Parameter	Range Tested	Base Case	HF Peak Location (m)	Stimulation Ratio	Leak Off Coefficient (×10^−3^ m/√min)	Productivity After 100 Days (m^3^·d^−1^·MPa^−1^)
Acid concentration (mol/L)	2.0–6.0	4	38–62	9.88–11.06	1.45–2.05	6.82–7.38
Injection rate (m^3^/min)	2.0–5.0	3.5	35–68	9.94–11.02	1.55–2.15	6.88–7.32
Temperature (°C)	60–110	85	42–58	10.15–10.82	1.58–1.98	6.95–7.25
Pressure (MPa)	35–75	55	40–60	10.20–10.70	1.52–1.88	6.98–7.18
Initial permeability (mD)	0.4–1.6	0.8	38–62	9.52–11.32	1.10–2.60	6.42–7.82

**Table 3:** Comparison of conventional fracturing and prepad acid fracturing (full/half removal).

Fracturing Method	Fracture Length (m)	Average Width (mm)	Stimulation Ratio	Initial Productivity (m^3^·d^−1^·MPa^−1^)	Productivity After 100 Days (m^3^·d^−1^·MPa^−1^)
Conventional hydraulic fracturing	175	4.41	9.09	6.48	6.03
Prepad acid fracturing (full removal)	162	3.98	10.54	7.51	7.14
Prepad acid fracturing (half removal)	162	3.98	9.97	7.11	6.64

**Table 4:** Basic reservoir petro physical parameters.

Parameter	Value	Unit
Burial depth	3000.0	m
Pay zone thickness	26.5	m
Initial porosity	8.0	%
Original permeability	0.8	mD
Fast-reacting mineral content	6.0	%
Slow-reacting mineral content	94.0	%
Formation pressure	55.0	MPa
Reservoir drainage radius	500.0	m

**Table 5:** Pumping schedule.

	Fluid Type	Volume (m^3^)	Injection Rate (m^3^/min)	Proppant Volume (m^3^)
1	Acid	20.0	3.5	0
2	Acid	20.0	3.5	0
3	Fracturing fluid	15.0	4.0	0
4	Fracturing fluid	18.0	4.0	1.8
5	Fracturing fluid	20.0	4.0	2.4
6	Fracturing fluid	22.0	4.0	3.8
7	Fracturing fluid	20.0	4.0	4.6

**Table 6:** Acid–rock reaction kinetic parameters.

Reaction	Reaction Rate Constant (mol·m^−2^·s^−1^·(mol·m^−3^)^−1^)	Activation Energy (J·mol^−1^)	Stoichiometric Coefficient
HF → Mineral I	0.127	6800	27
HF → Mineral II	2.32 × 10^−3^	1150	6
HF → Mineral III	2.0 × 10^−4^	2500	6
H_2_SiF_6_ → Mineral I	5.0 × 10^−5^	3600	1

**Table 7:** Parameters for fracturing fluid damage and acid cleanup.

Parameter	Symbol	Value	Unit
Original reservoir permeability	*k*	0.8	mD
Reservoir permeability after fracturing fluid damage	*k_d_*	0.45	mD
Fracturing fluid filter cake permeability	*k_c_*	8 × 10^−4^	mD
Propped fracture permeability	*k_f_*	1 × 10^5^	mD
Fracture permeability after fracturing fluid damage	*k_fd_*	5 × 10^4^	mD
Reservoir permeability after acid cleanup	*k′*	0.85	mD
Filter cake permeability after acid cleanup	*k_c_′*	0.1	mD
Fracture permeability after acid cleanup	*k_fd_′*	7 × 10^4^	mD

## Data Availability

All data, models, or code generated or used during the study are available from the corresponding author by request.

## Nomenclature

**Symbol**	**Description**	**Unit**
*C*	Acid concentration (HBF_4_ or HF)	mol/m^3^
*C_D_*	Dimensionless acid concentration	—
*C_f_* (x,t)	Acid concentration in the fracture	mol/m^3^
*C_HF_*	HF concentration	mol/m^3^
*C_mj_*	Concentration of mineral category *j*	mol/m^3^
*C_mj_*_,0_	Initial concentration of mineral category *j*	mol/m^3^
*C_PA_*	HBF_4_ concentration	mol/m^3^
*C*_0_	Initial acid concentration	mol/m^3^
*C_L_*	Leak-off coefficient	m/√min
*C_L,i_*	Leak-off coefficient for cell *i*	m/√min
C*_L,total_*	Overall leak-off coefficient	m/√min
*Da*	Damköhler number (leak-off zone)	—
*Da*_f_	Damköhler number (fracture)	—
*f_gel_*(*C_HF_*,t)	Gel cleanup factor	—
*H*	Fracture height	m
*k*	Permeability	mD
k_0_	Initial permeability	mD
*k_avg_*	Average permeability of leak-off zone	mD
*khyd*	HBF_4_ hydrolysis rate constant	s^−1^
*kj*	Reaction rate constant for mineral *j*	mol/(m^2^·s·(mol/m^3^))
*L*	Fracture length	m
*L_f_*	Leak-off depth	m
*M_j_*	Molar mass of mineral *j*	kg/mol
*N*	Number of fracture cells	—
*M*	Number of leak-off depth cells	—
*N_ac_*	Acid capacity number	—
Δ*P*	Pressure difference across fracture wall	Pa
*R_PA_*	HBF_4_ hydrolysis rate to HF	mol/(m^3^·s)
*R_r,HF_*	HF consumption rate by mineral reactions	mol/(m^3^·s)
S*_j_*	Specific surface area of mineral *j*	m^2^/m^3^
*t*	Time	s or min
*t_D_*	Dimensionless time	—
*u*	Acid flow velocity within fracture	m/s
*u_D_*	Dimensionless velocity	—
*u_la_*	Leak-off velocity	m/s
*w*	Fracture width	m
*x*	Coordinate along fracture direction	m
*x_D_*	Dimensionless distance (fracture direction)	—
*y*	Coordinate along leak-off depth direction	m
*y_D_*	Dimensionless distance (leak-off direction)	—
α	Cementation exponent	—
Δ*x_i_*	Length of fracture cell *i*	m
Δ*y*	Leak-off depth cell length	m
*ε*	Convergence tolerance	—
*ϕ*	Porosity	—
*ϕ*_0_	Initial porosity	—
*μ*	Fluid viscosity	Pa·s
*ν_j_*	Stoichiometric coefficient for mineral *j*	—
*ρ_j_*	Density of mineral *j*	kg/m^3^
